# Network of clinically-relevant lncRNAs-mRNAs associated with prognosis of hepatocellular carcinoma patients

**DOI:** 10.1038/s41598-020-67742-8

**Published:** 2020-07-07

**Authors:** Lee Jin Lim, Yu Jin, Henry Yang, Alexander Y. F. Chung, Brian K. P. Goh, Pierce K. H. Chow, Chung Yip Chan, William K. Blanks, Peng Chung Cheow, Ser Yee Lee, Tony K. H. Lim, Samuel S. Chong, London L. P. J. Ooi, Caroline G. Lee

**Affiliations:** 1 Department of Biochemistry, Yong Loo Lin School of Medicine, National University of Singapore, Singapore, Singapore; 2 Division of Cellular and Molecular Research, Humphrey Oei Institute of Cancer Research, National Cancer Centre Singapore, Level 6, Lab 5, 11 Hospital Drive, 169610 Singapore, Singapore; 3 NUS Graduate School for Integrative Sciences and Engineering, National University of Singapore, Singapore, Singapore; 4 Cancer Science Institute of Singapore, National University of Singapore, Singapore, Singapore; 5 Department of Hepato-Pancreato-Biliary and Transplant Surgery, Singapore General Hospital, Singapore, Singapore; 6 Duke-NUS Graduate School, Singapore, Singapore; 7 Department of Surgical Oncology, National Cancer Centre Singapore, Singapore, Singapore; 8 The University of North Carolina, Chapel Hill, USA; 9 Department of Pathology, Singapore General Hospital, Singapore, Singapore; 10 Department of Pediatrics, Yong Loo Lin School of Medicine, National University of Singapore, Singapore, Singapore

**Keywords:** Hepatocellular carcinoma, Long non-coding RNAs

## Abstract

Long non-coding RNAs (lncRNAs) are often aberrantly expressed in Hepatocellular Carcinoma (HCC). We hypothesize that lncRNAs modulate HCC prognoses through differential deregulation of key lncRNAs affecting important gene network in key cancer pathways associated with pertinent clinical phenotype. Here, we present a novel approach integrating lncRNA-mRNA expression profiles with clinical characteristics to identify lncRNA signatures in clinically-relevant co-expression lncRNA-mRNA networks residing in pertinent cancer pathways. Notably one network, associated with poorer prognosis, comprises five up-regulated lncRNAs significantly correlated (|Pearson Correlation Coefficient|≥ 0.9) with 91 up-regulated genes in the cell-cycle and Rho-GTPase pathways. All 5 lncRNAs and 85/91 (93.4%) of the correlated genes were significantly associated with higher tumor-grade while 3/5 lncRNAs were also associated with no tumor capsule. Interestingly, 2/5 lncRNAs that are correlated with numerous genes in this oncogenic network were experimentally shown to up-regulate genes involved in cell-cycle and transcriptional regulation. Another network comprising 4 down-regulated lncRNAs and 8 down-regulated metallothionein-family genes are significantly associated with tumor invasion. The identification of these key lncRNAs signatures that deregulate important network of genes in key cancer pathways associated with pertinent clinical phenotype may facilitate the design of novel therapeutic strategies targeting these ‘master’ regulators for better patient outcome.

## Introduction

Hepatocellular Carcinoma (HCC) is the sixth commonest cancer and fourth most common cause of cancer deaths worldwide^1^. Factors reported to increase the risks for HCC, include viral Hepatitis B and C, non-alcoholic fatty liver diseases and non-alcoholic steatohepatitis^2^. Patients diagnosed with late-stage HCC have a 5-year survival rate of less than 5%, but increases to 70.1–77.2% if patients are diagnosed with HCC at an early stage. However, patients with early stage HCC who undergo resection for treatment are still at a high risk for recurrence within 5 years^3^. Hence, it is important to identify key molecules that play roles in improving the prognosis of HCC patients.

Long non-coding RNAs (LncRNAs) are RNAs that are > 200 nucleotides in length and do not encode for protein. LncRNAs share many similar characteristics with mRNAs. Both are transcribed by RNA polymerase II, spliced, capped at the 5′end and poly(A) tail at the 3′end^4^. Compared to other non-coding RNAs, the function of lncRNAs are less well-known due to poor conservation of sequence, hindering sequence-based function prediction^5^. However, researchers have shown that lncRNAs are able to regulate gene expression at every stage of the life cycle of a gene^6^. Deregulation of lncRNA’s expression has been reported in various cancers including HCC. A recent review highlighted that a large number of lncRNAs (e.g. HULC^7,8^, MALAT-1^9–11^ and UCA1^12,13^) are deregulated in HCC patients^14^. Nonetheless, most current studies mainly examine each lncRNAs individually. As different lncRNAs may function together to play roles in tumorigenesis, metastasis or even modulating patient outcome, it is thus worthwhile to identify and characterize combinations of lncRNAs that have potential to function synergistically.

**Figure 1:**
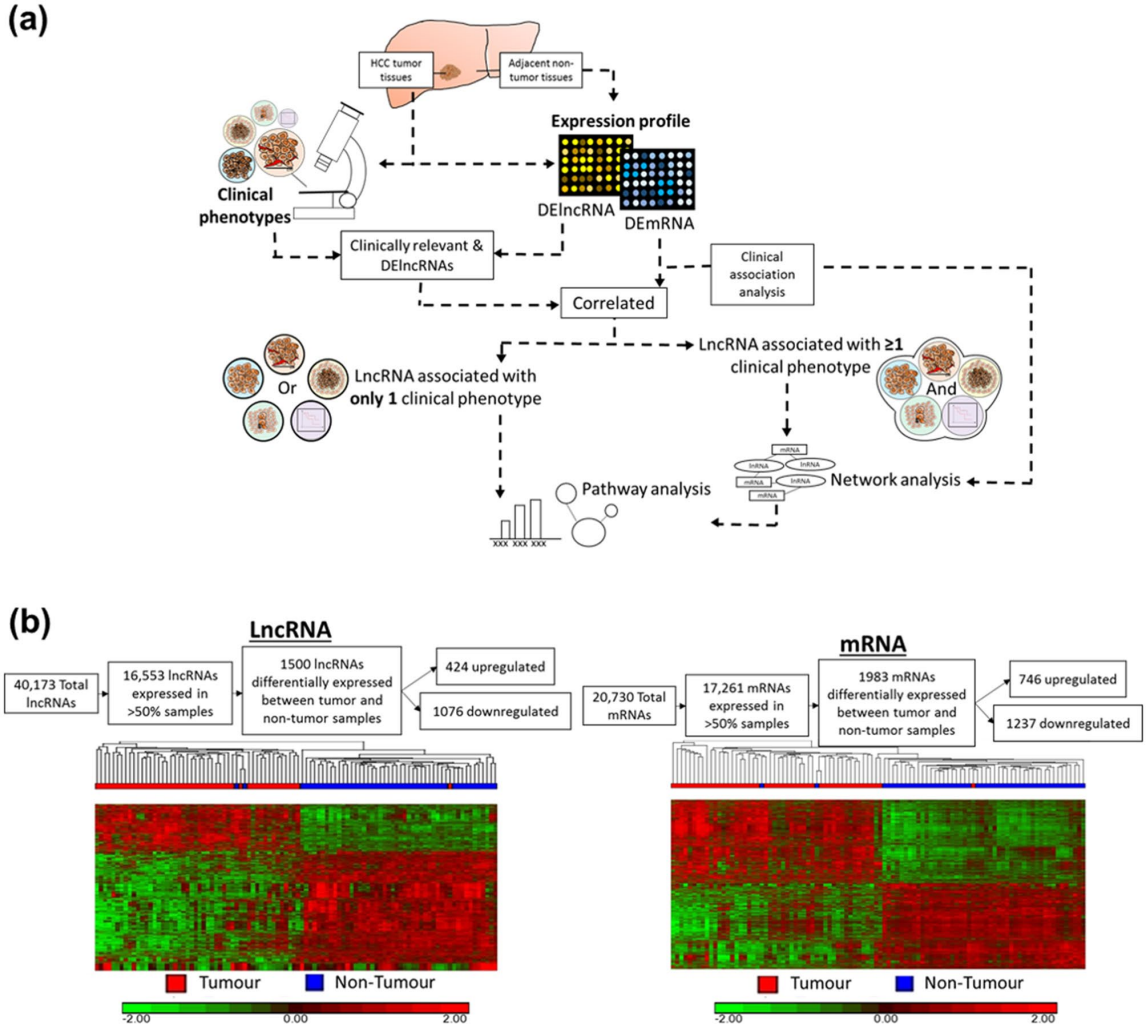
(**a**) Schematic overview of the strategy used to study pathways associated with clinically relevant lncRNAs and its potential regulated genes. LncRNA and mRNA expression profile are performed on HCC tumor tissues and adjacent non-tumor tissues. Clinical phenotypes of HCC tumor tissues are used for clinical association studies. Only differentially expressed (DE) and clinically relevant lncRNAs are included for further analysis. DEmRNAs that are correlated with clinically relevant DElncRNAs are included for further analysis. Clinical association of DEmRNAs are also identified. Two different analysis are carried out on clinically relevant lncRNAs-mRNAs pairs. Among correlated pairs, lncRNAs that are associated with only 1 clinical phenotype are selected for pathway analysis to identify potential prognostic markers. LncRNAs that are associated with more than 1 clinical phenotypes are selected for network analysis, followed by pathway analysis. Clinical association of DEmRNAs are also considered in the network analysis. Colored circles represent 5 groups of clinical phenotypes in this study: Tumor properties in orange, Tumor capsule in yellow, Tumor grade in blue, Tumor invasion in green, Patient overall survival in purple. (**b**) Differentially expressed lncRNAs and mRNAs between tumor (T) and non-tumor (NT) samples. Top: Workflow shows the number of lncRNAs/mRNAs at different stages of the workflow. Bottom: heatmap of the differentially expressed lncRNAs/mRNAs in T and NT samples. Each column in the heatmap represent the patient tissues samples types: red in column: T; blue in column: NT. Each row in the heatmap represents lncRNA/mRNA: red: Upregulation; green: Downregulation.

In this study, we adopted a novel approach to identify and characterize combinations of clinically relevant, deregulated lncRNAs with potential to act synergistically by integrating lncRNA, mRNA expression profiles with clinical characteristics (Fig. 1a). Through profiling of both lncRNA and mRNAs, significantly deregulated lncRNAs and mRNAs were identified. Clinical relevance of these deregulated lncRNAs and genes were then determined through association analyses with various clinical characteristics. Genes that are co-expressed with lncRNAs were identified through “Guilt by Association”^15,16^ and the pertinent cancer pathways that these co-expressed genes reside were then determined to gain insights into pathways deregulated by lncRNAs through their co-expressed mRNA^17–23^. Networks of clinically-relevant, deregulated lncRNAs with their correlated clinically relevant, deregulated mRNA were then generated and the pathways enriched by the constituent genes in the network were determined. Two different categories of pathways were generated. The first comprises networks of lncRNAs associated with one or more phenotype while the other comprises lncRNAs that are uniquely associated with only a single clinical phenotype.

With this strategy, combinations of lncRNAs that can serve as potential master regulator of genes in clinically-relevant, co-expression lncRNA-mRNA networks in pertinent cancer pathways were identified.

## Results

### Differentially expressed lncRNAs and mRNAs in HCC tissues

A genome-wide expression profile of the tumor versus non-tumorous liver in 49 HCC patients was performed to identify differentially expressed lncRNAs and mRNAs. A total of 40,173 lncRNAs and 20,730 mRNAs were interrogated using the Arraystar Human LncRNA Array V4.0. 16,553 lncRNA transcripts and 17,261 mRNA transcripts are expressed in more than 50% of the total samples. Using FDR corrected *P* value of < 0.05 and absolute fold change of > 2.0, 1,500 (424 up-regulated and 1,076 down-regulated) lncRNA and 1,983 (746 up-regulated and 1,237 down-regulated) mRNA transcripts are differentially expressed between HCC tumour tissues and adjacent non-tumourous tissues (Fig. 1b). Unsupervised hierarchical clustering is able to differentiate the HCC tumour from adjacent non-tumourous tissues, suggesting the expression of lncRNAs and mRNAs are different between HCC tumour and the adjacent non-tumourous tissues (Fig. 1b).

### Co-expression between differentially expressed lncRNAs and mRNAs

**Figure 2:**
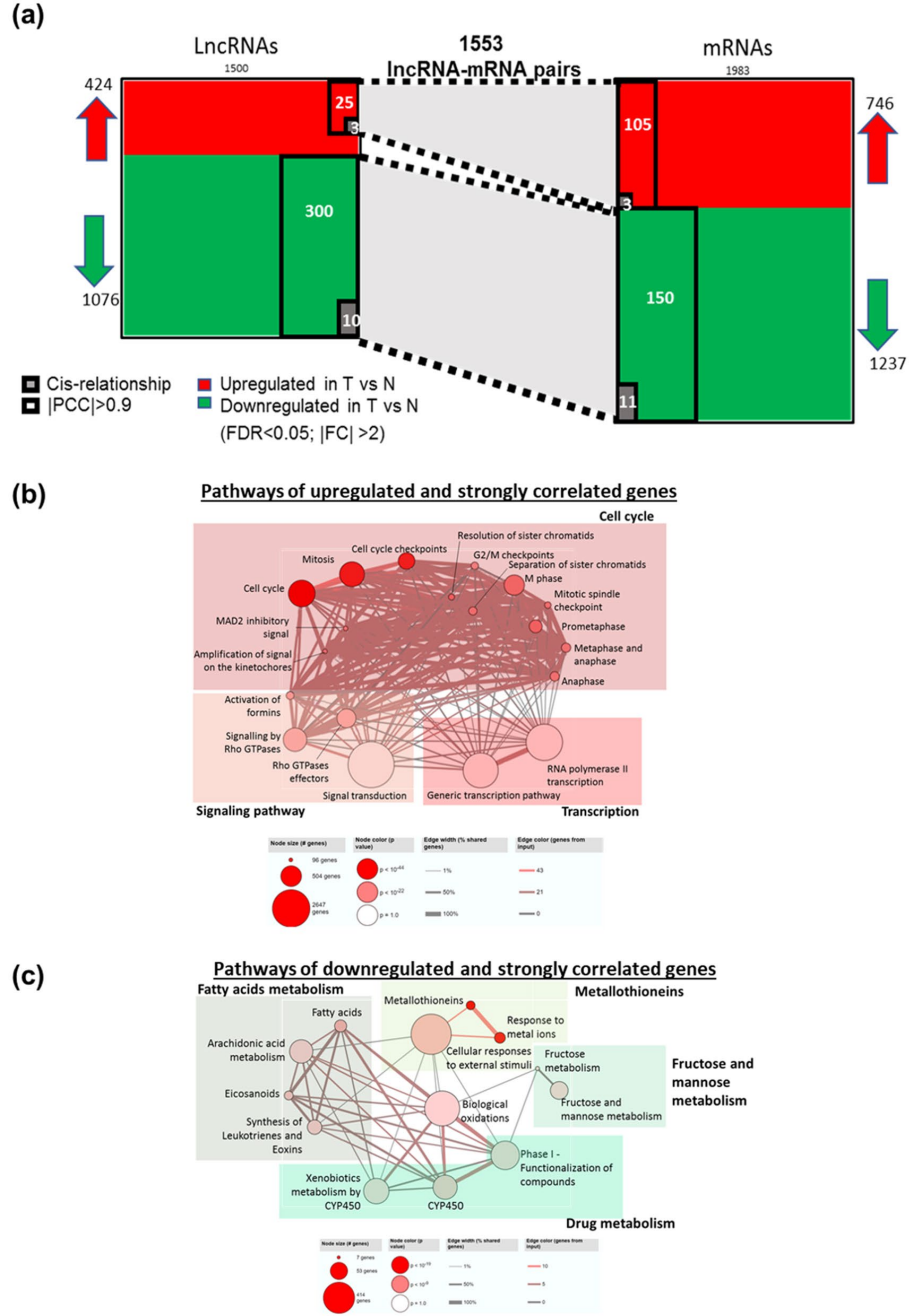
(**a**) Summary of correlation between differentially expressed lncRNAs and mRNAs. Red: Upregulation in T versus NT; green: downregulation in T versus NT. Strong positive correlation with |R|≥ 0.9 is shown as dotted black lines with light grey background. Number of differentially expressed lncRNAs/mRNAs that has |R|≥ 0.9 are shown within the thick black boxes with white fonts. Small black boxes with grey background represents number of correlations that is also in cis-relationship. (**b**) Pathways of upregulated strongly correlated genes are mainly related to cell cycle, signaling pathways and transcription pathways. (**c**) Pathways of downregulated strongly correlated genes are mainly related to fatty acid metabolism, drug metabolism, fructose and mannose metabolisms and metallothioneins.

The “Guilt-by-association” model was employed to understand the pathways enriched with deregulated protein-coding genes that are co-expressed with the deregulated lncRNAs^15,16^. Pearson correlation analyses was performed on 1,500 differentially expressed lncRNAs and 1983 differentially expressed mRNAs transcripts to identify significantly co-expressed lncRNA-mRNA pairs. With a |R|≥ 0.90 threshold, a total of 1553 lncRNAs-mRNA co-expressing pairs, comprising 339 lncRNA and 269 mRNA transcripts were identified. Notably, none of the 1553 co-expression pairs were inversely correlated. The co-expressing pairs comprise 28 up-regulated lncRNAs whose expression correlated with 108 up-regulated mRNAs and 311 down-regulated lncRNAs whose expression correlated with 161 down-regulated mRNAs (Fig. 2a). Among the 1553 co-expression pairs, 14 pairs are identified as having a *cis* relationship (defined as lncRNAs co-expressed with its nearest protein coding genes). To explore the functions of the genes strongly correlated with the lncRNAs, pathway analysis was carried out on the 108 up-regulated mRNAs and 161 down-regulated mRNAs transcripts using ConsensusPathDB database, respectively^24,25^. Our results showed that the up-regulated associated mRNAs were mostly enriched in cell cycle pathways, Rho GTPases signaling pathways and transcription pathways (Fig. 2b), while down-regulated associated mRNAs were mostly enriched in metallothionein, fructose and mannose metabolism, fatty acid metabolism and drug metabolism (Fig. 2c).

### Association of strongly correlated lncRNAs with clinicopathological features

**Figure 3:**
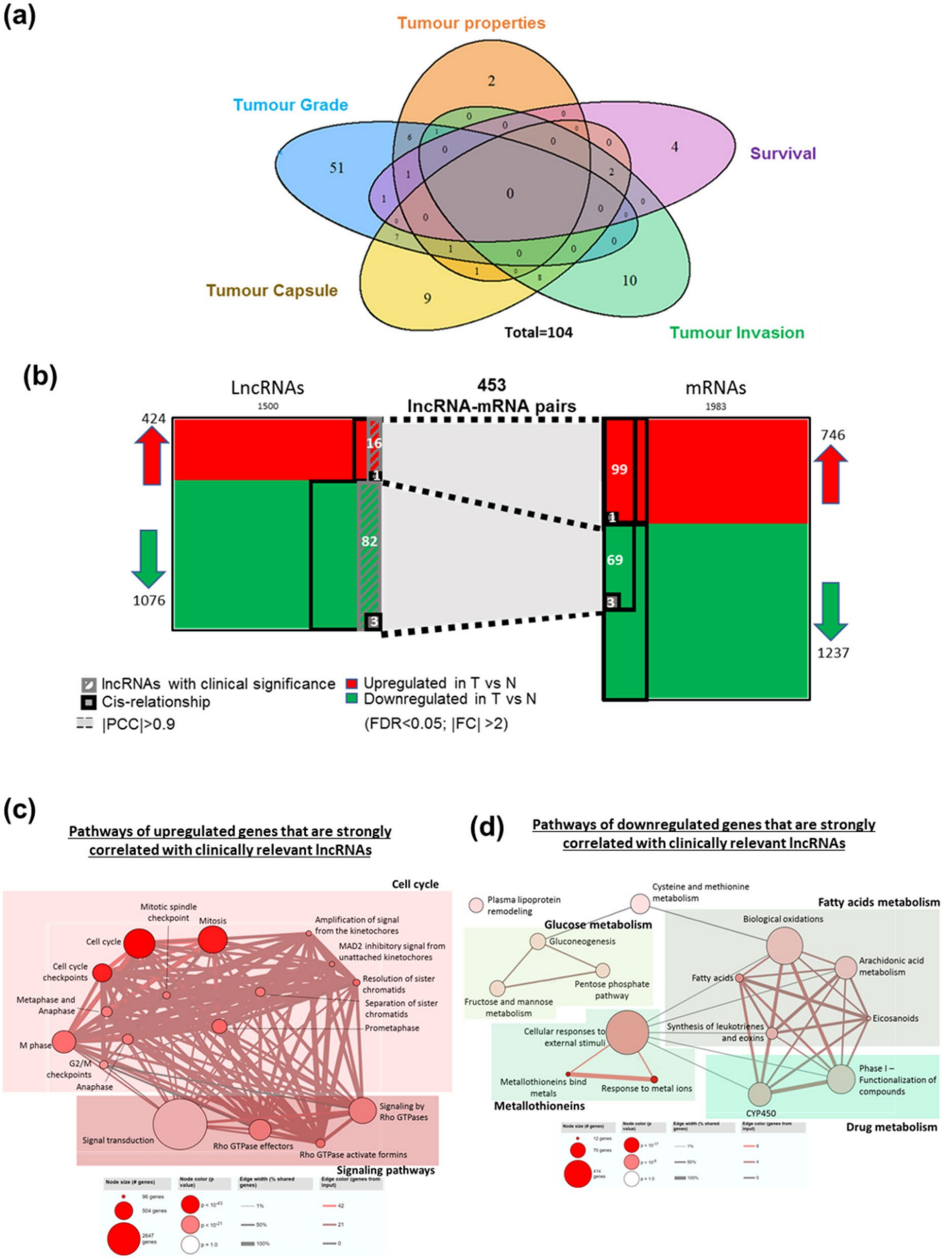
(**a**) Venn diagram shows the number of clinically relevant lncRNAs, which is defined as lncRNAs that is differentially expressed in T versus NT and also differentially expressed in poor clinical characteristic versus good clinical characteristic. Their expressions in both T versus NT and poor clinical characteristic versus good clinical characteristic are in the same direction and significant. Blue: Edmondson Grade; orange: tumor properties (Includes tumor size, tumor stage and vascularization); purple: overall survival; green: tumor invasion; yellow: tumor capsule (includes encapsulation and degree of encapsulation). (**b**) Summary of strong correlation between clinically relevant lncRNAs and differentially expressed mRNAs. Red: upregulation in T versus NT; green: downregulation in T versus NT. Strong positive correlation with |R|≥ 0.9 is shown as dotted black lines with light grey background. Number of differentially expressed lncRNAs/mRNAs that has |R|≥ 0.9 are shown within the thick black boxes with white fonts. Number of clinically relevant lncRNAs that is also strongly correlated with mRNA are shown in grey box with lines. Small black boxes with grey background represents number of correlation that is also in cis-relationship. (**c**) Pathways of upregulated clinically relevant and strongly correlated genes are mainly related to cell cycle and signaling pathways. (**d**) Pathways of downregulated clinically relevant and strongly correlated genes are mainly related to plasma lipoprotein remodeling, glucose, amino acids, fatty acids and drug metabolism, as well as metallothioneins.

To explore clinical significance of the 339 strongly correlated lncRNAs, clinical phenotypes were categorized into five groups, namely, tumour properties (cancer stage, tumour size and vascularization), tumor grade, tumour capsule (encapsulation and degree of encapsulation), tumour invasion and overall survival status (Fig. 3a). A total of 104 strongly correlated lncRNAs were identified as clinically significant, deregulated lncRNAs as their expression is de-regulated in the tumors of HCC patients and they are also significantly associated with clinical characteristics in at least one clinical group (Fig. 3a). 17 of these were potential oncogenic lncRNAs with their high expression in the tumors being associated with poorer clinical characteristics while 87 were potential tumor suppressor lncRNAs since high expression of these lncRNAs in tumors were associated with better clinical characteristics. These clinically associated lncRNAs were found to be significantly and positively correlated (|PCC|≥ 0.9) with 172 mRNAs (100 upregulated mRNAs and 72 downregulated mRNAs), generating 453 deregulated, clinically associated lncRNAs–mRNA co-expression pairs (Fig. 3b). Pathway analyses revealed that the potential oncogenic lncRNAs were significantly enriched with genes in the cell-cycle and Rho GTPases signaling pathway (Fig. 3c) while the potential tumor suppressor lncRNAs were significantly enriched with genes in the metallothionein family, glucose metabolism, cysteine and methionine metabolism, fatty acids metabolism, drug metabolism and plasma lipoprotein remodeling (Fig. 3d).

### Analyses of clinically associated lncRNA-mRNA co-expression networks

Cytoscape^26^ was employed to further analyze the relationship of the 453 deregulated, clinically associated lncRNA-mRNA co-expression pairs. A total of 37 network were generated, including 4 in *cis* relationship (Figure S1). Notably, 81% of the correlated mRNAs have the same clinical significance as their correlated lncRNAs. Ten of these networks were potentially oncogenic (Figure S1a) while 27 were potentially tumor suppressive (Figure S1b).

**Figure 4:**
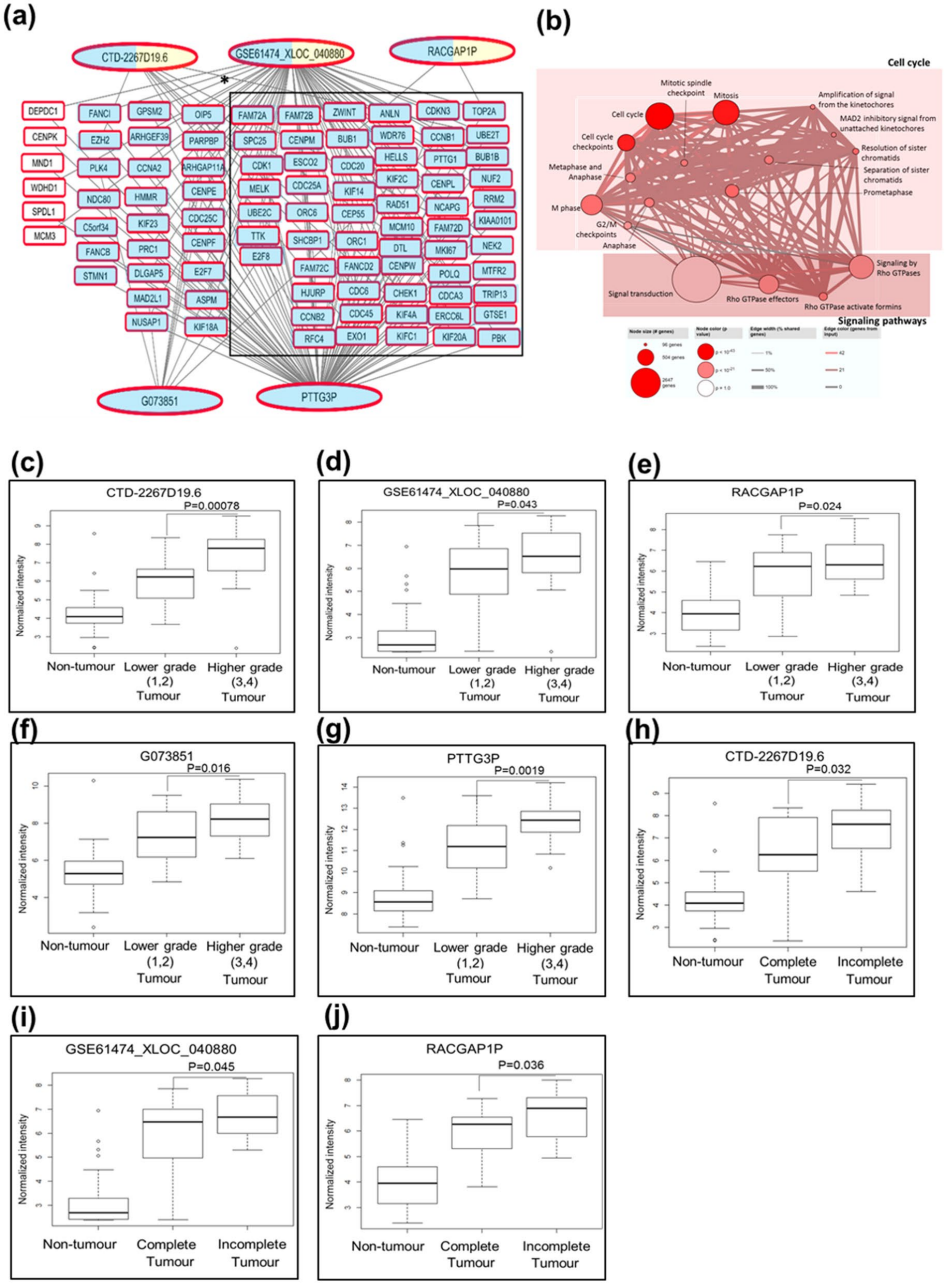
(**a**) Network containing potential oncogenic lncRNAs that could be master regulators of 91 cell cycle genes. High expression of the 5 potential oncogenic lnRNAs (G073851, PTTG3P, RACGAP1P, GSE61474_XLOC_040880 and CTD-2267D19.6) and 85/91 mRNAs are associated with poor differentiation of tumor. High expression of 3/5 potential oncogenic lncRNAs (RACGAP1P, GSE61474_XLOC_040880 and CTD-2267D19.6) are associated with incomplete tumor capsule. Oval shape: lncRNAs; Square: mRNAs; black lines: Strong correlation. Outline of oval and square represents lncRNAs/mRNAs expression in T versus NT; red: upregulation; colours within oval/square represents the clinical significance of lncRNAs/mRNAs. Blue background: Edmondson Grade; yellow background: tumor capsule (includes encapsulation and degree of encapsulation); *: clinical significance with FDR < 0.05. The network was drawn using Cytoscape software (Version 3.5.1) (https://cytoscape.org/). (**b**) Pathways of the 91 strongly correlated genes. (**c**–**g**) Boxplots show expression of lncRNAs in adjacent non-tumor, lower grade tumor and higher grade tumor. (**h**–**j**) Boxplots show expression of lncRNAs in adjacent non-tumor, tumor with capsule and tumor without capsule.

Potential clinically-relevant master lncRNA regulator(s), were defined as the minimum number of lncRNAs in a large network that is not only associated with key clinical characteristics as well as a large number of significantly (|PCC|≥ 0.9) correlated mRNAs, > 80% of these correlated mRNAs in the network were also associated with the same clinical characteristics as the lncRNAs, and the co-expressed mRNAs reside in pertinent cancer pathways. Significantly, one oncogenic network comprising 5 clinically-relevant potentially oncogenic lncRNAs (G073851 (seqname: T314711^27^), PTTG3P (seqname: NR_002734), RACGAP1P(seqname: NR_026583), GSE61474_XLOC_040880 (seqname: GSE61474_TCONS_00220963^28^) and CTD-2267D19.6 (seqname: ENST00000602403)) correlated with 91 potentially oncogenic mRNAs (Fig. 4a) residing in the cell cycle and Rho GTPases signaling pathways (Fig. 4b) was identified. Interestingly, the 91 genes co-expressed with these 5 master/nodal/key lncRNAs reside in the same cell-cycle and Rho GTPases signaling pathways (Fig. 4b) as the 100 genes co-expressed with the 17 clinically relevant lncRNAs (Fig. 3c) highlighting that only 5 lncRNAs may be sufficient to modulate these 2 key cancer pathways. The other 9 genes that are significantly co-expressed with the other 12 clinically-relevant lncRNAs were not significantly associated with any pertinent cancer pathways. Higher tumor expression of 5 lncRNAs was found to be associated with poorer prognosis of higher tumor grade (Fig. 4c–g). Similarly, 85/91 co-expressed mRNAs of these 5 lncRNAs were also associated with higher tumor grade (Fig. 4a). Additionally, higher tumor expression of 3/5 lncRNAs (RACGAP1P, GSE61474_XLOC_040880 and CTD-2267D19.6) was found to be associated with incomplete tumour encapsulation which may lead to increased risk of metastasis^29^ (Fig. 4h–j). These 5 lncRNAs thus have the potential to serve as master regulators of genes associated with poorer prognosis of HCC patients.

To better understand the role of key/nodal/master lncRNAs in the oncogenic network associated with tumor grade/tumor capsule (Fig. 4a), genes deregulated by GSE61474_XLOC_040880 (lncRNA-GSE) and CTD-2267D19.6 (lncRNA-CTD) were determined experimentally, since these lncRNAs were significantly correlated with a large number of genes in the network, and their expression were successfully validated in 59 patients with real-time RT-PCR (Figure S2). Although PTTG3P was also highly up-regulated and correlated with many genes, it was not selected for further analyses because the probes in the microarray bound to both the PTTG3P lncRNA and PTTG1 mRNA and we were unable to validate the lncRNA expression with real-time RT-PCR using PTTG3P-specific primers.

**Figure 5:**
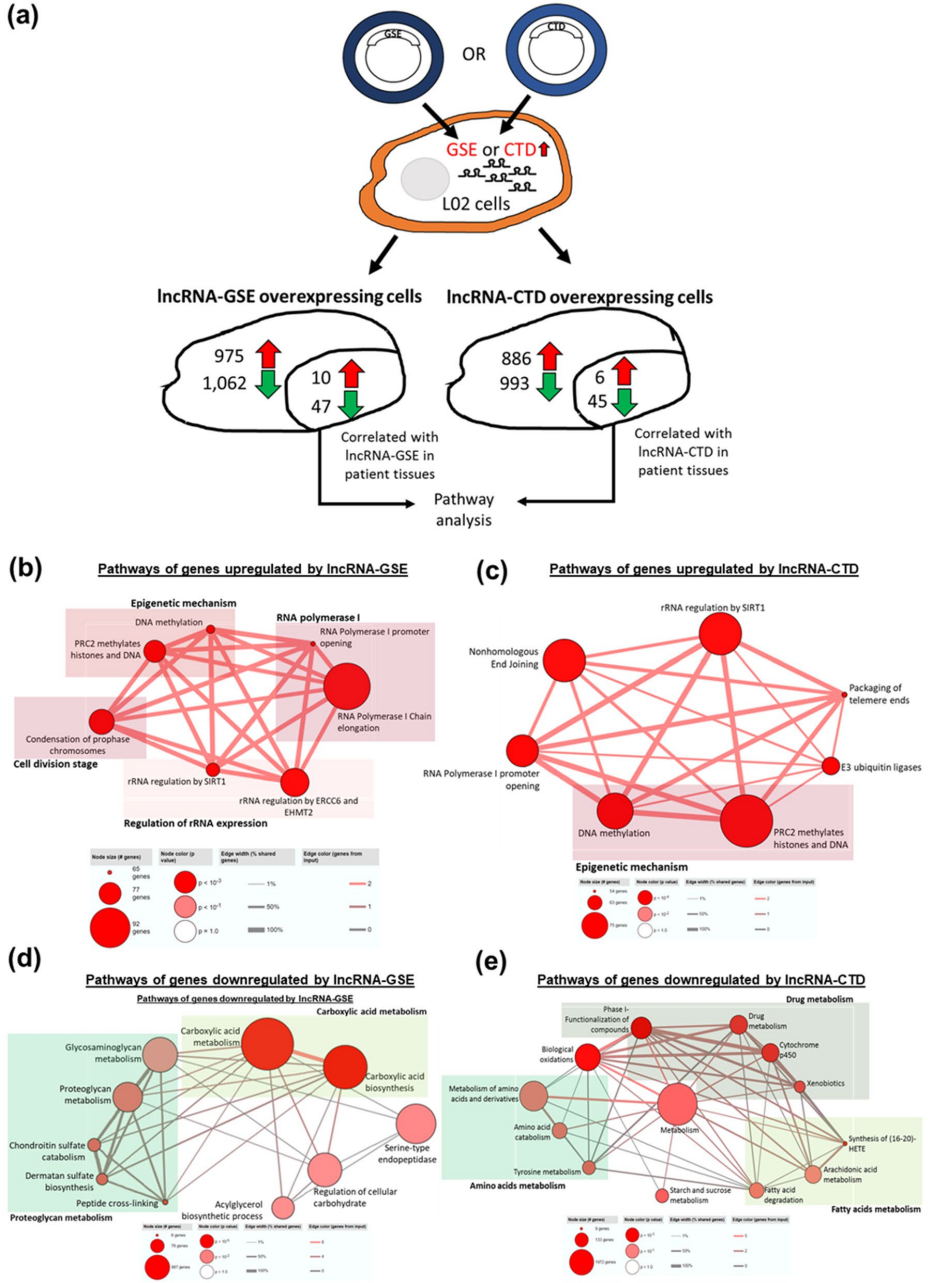
(**a**) Figure shows the strategy used to identify deregulated genes of lncRNA-GSE and lncRNA-CTD in L02 cells. LncRNA-GSE and lncRNA-CTD were transfected into L02 cells respectively. RNA sequencing was then performed on lncRNA-GSE overexpressing cells and lncRNA-CTD overexpressing cells to identify genes deregulated by lncRNA-GSE and lncRNA-CTD. Genes that are differentially expressed by |FC|≥ 2 in lncRNA-GSE overexpressing cells versus control or lncRNA-CTD overexpressing cells versus control were selected for further analysis. The differentially expressed genes that are correlated with lncRNA-GSE/lncRNA-CTD (|PCC|≥ 0.6) in the same direction in patient microarray dataset were included for pathway analysis. Red arrow: upregulated mRNAs; green arrow: Downregulated mRNAs. (**b**) Pathways of genes upregulated by lncRNA-GSE are mainly related to epigenetic mechanism, RNA polymerase I activity, cell division stage and regulation of rRNA expression. (**c**) Pathways of genes upregulated by lncRNA-CTD are involved in epigenetic mechanism, RNA polymerase I activity, Nonhomologous end joining, rRNA regulation, packaging of telomere ends and E3 ubiquitin ligases. Each line represents the common genes shared between the pathways. (**d**) Pathways of genes downregulated by lncRNA-GSE are related to carboxylic acid metabolism, proteoglycan metabolism, aclyglycerol biosynthetic process, regulation of cellular carbohydrates and serine-type endopeptidase. (**e**) Pathways of genes downregulated by lncRNA-CTD are mainly involved in amino acids, starch and sucrose, fatty acids and drug metabolism.

These 2 lncRNAs were transfected into LO2, an immortalized liver cell line and RNA sequencing was performed (Figure S3). Genes, whose expression were altered (by ≥ 2 times) when the lncRNAs were over-expressed, and significantly correlated (|PCC|≥ 0.6) in the appropriate direction with lncRNA-GSE or lncRNA-CTD in the HCC patients were selected for pathway analyses (Fig. 5a). The 10 genes, which were up-regulated in cells that over-expressed lncRNA-GSE and correlated with lncRNA-GSE in the patients were found to be associated with cell division/cell-cycle and transcription regulation (RNA polymerase I (Pol I) activity, epigenetic mechanism and regulation of rRNA expression) (Fig. 5b). Similarly the 6 genes whose expression were increased in lncRNA-CTD overexpressing cells were found to be associated with protein degradation (E3 ubiquitin ligases), but mainly in the transcriptional regulation (including RNA Pol I activity, epigenetic mechanism, nonhomologous end joining, packaging of telemore ends, regulation of rRNA expression) pathways (Fig. 5c). On the other hand, genes that are down-regulated in either lncRNA-GSE or lncRNA-CTD overexpressing cells and correlated with these lncRNAs in the patients were found to be mainly associated with metabolism (Figs. 5d,e). The 47 genes whose expression is attenuated in lncRNA-GSE overexpressing cells and correlated in HCC patients were found to reside mainly in proteoglycan and carboxylic acid metabolism pathway (Fig. 5d) while the 45 down-regulated genes in lncRNA-CTD overexpressing cells and correlated in HCC patients are mainly in the drug, fatty acid and amino acid metabolism (Fig. 5e). Hence, these 2 master regulators mainly up-regulate genes involved in transcription regulation while down-regulating genes involved in metabolism.

**Figure 6:**
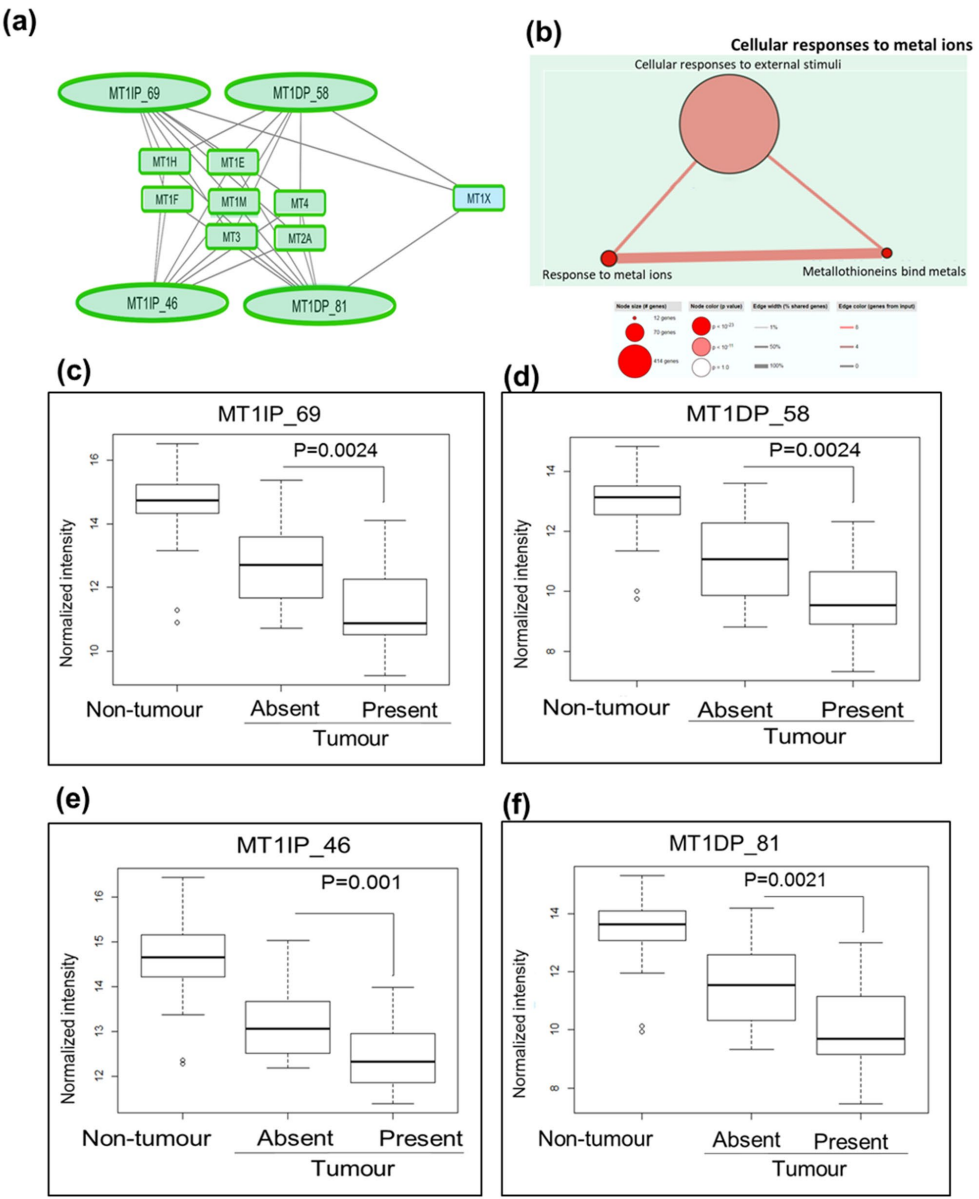
(**a**) Network containing potential tumor suppressing lncRNAs that could be master regulators of 8 metallothionein genes. Low expression of the 4 potential tumor suppressing lnRNAs (MT1DP isoforms (NR_027781 and NR_003658)) and MT1IP isoforms (NR_003669 and NR_104046)) and 7 mRNAs are associated with tumor invasion. Oval shape: lncRNAs; Square: mRNAs; Black lines: Strong correlation. Outline of oval and square represents lncRNAs/mRNAs expression in T versus NT; green: downregulation; colours within oval/square represents the clinical significance of lncRNAs/mRNAs. Green background: Tumor invasion. The network was drawn using Cytoscape software (Version 3.5.1) (https://cytoscape.org/). (**b**) Pathways of the 8 strongly correlated genes. (**c**–**f**) Boxplots show expression of lncRNAs in adjacent non-tumor, tumor without invasion and tumor with invasion.

On the other hand, clinically-relevant lncRNAs as master regulators of genes associated with tumor suppressive networks were less clear. Nonetheless, one tumor suppressive network comprising 4 tumour suppressor lncRNAs (MT1DP (seqname: NR_027781 and NR_003658) and MT1IP (seqname: NR_003669 and NR_104046)) and 8 co-expressed genes (Fig. 6a), enriched in the metallothionein family (Fig. 6b) was identified. Lower expression of all 4 tumour suppressor lncRNAs and 7/8 genes were found to be associated with higher invasive potential (Fig. 6c–f) suggesting that higher expression of these lncRNAs/genes may decrease risk of metastasis of HCC patients.

### Pathways of lncRNAs uniquely associated with specific clinical phenotypes

**Figure 7:**
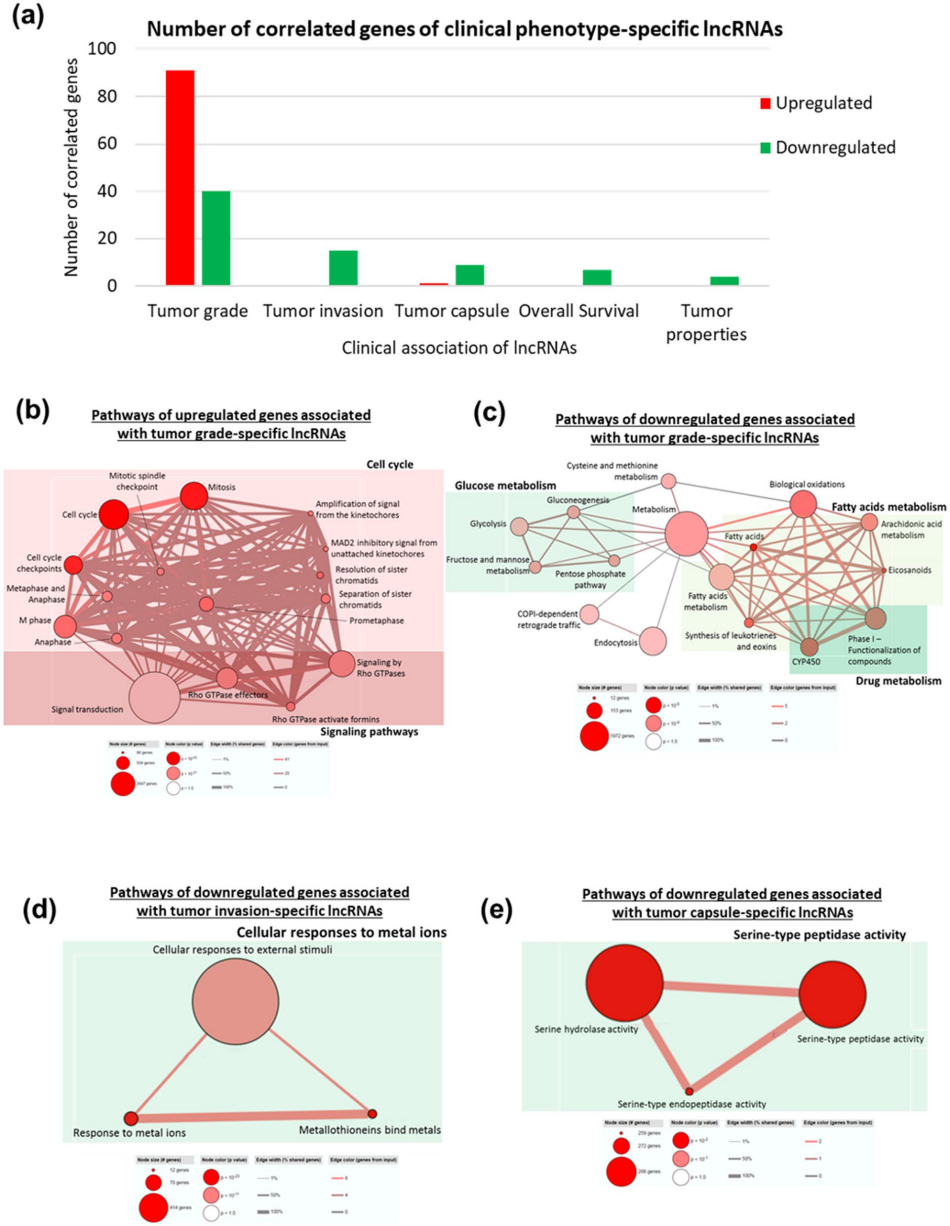
(**a**) Bar chart shows the number of genes correlated with lncRNAs specific to each clinical phenotypes. Red: upregulated mRNAs; green: downregulated mRNAs. (**b**) Pathways of genes correlated with upregulated lncRNAs specific to tumor grade are mainly related to DNA repair, cell cycle, p53 signaling pathways, viral infection and cellular senescence. (**c**) Pathways of genes correlated with downregulated lncRNAs specific to tumor grade are mainly related to fatty acid metabolism, glucose metabolism, drug metabolism and COP1-dependent retrograde traffic. (**d**) Pathways of genes correlated with downregulated lncRNAs specific to tumor invasion are mainly related to cellular response to metal ions. (**e**) Functional annotation shows that genes correlated with downregulated lncRNAs specific to tumor capsule are mainly involved in serine-type peptidase activity.

As lncRNAs that are uniquely associated with specific clinical characteristics may modulate genes that play important roles in that specific characteristic, we thus focused on the 51 (7 up- and 44 down-regulated), 9 (1 up- and 8 down-regulated), 10 (all down-regulated) and 4 (all down-regulated) lncRNAs that are uniquely associated with tumor grade, tumor capsule, tumor invasion and overall survival, respectively (Fig. 7a). Genes significantly correlated (|PCC|≥ 0.9) with these lncRNAs (Fig. 7a) were identified. Similar to what was observed earlier (Fig. 3c), the 91 up-regulated genes significantly correlated with 51 lncRNAs that are significantly associated with tumor grade were found to be enriched in the cell cycle and Rho GTPases signaling pathways (Table S1a, Fig. 7b). The 40 down-regulated, tumor grade-specific lncRNA-correlated genes were enriched in metabolism including fatty acid/arachidonic acid, drug and glucose metabolism, as well as COPI-dependent retrograde transport (Table S1a, Fig. 7c). The 15 down-regulated genes that correlated with the 10 down-regulated, tumor invasion-specific lncRNAs were found to be enriched in cellular responses to external stimuli (e.g. metal ions) (Table S1b, Fig. 7d). The 9 down-regulated genes that are strongly correlated with the 9 tumor capsule-specific lncRNAs are enriched in pathways related to peptidase and hydrolase activities (Table S1c, Fig. 7e). The genes associated with overall survival and tumor properties specific lncRNAs were not significantly enriched in any specific pathways (Table S1d and e).

### Data in this study is generally consistent with data from TCGA

To evaluate if the observations of this study is consistent in another cohort of patients, we analyzed the RNA sequencing data of 361 HCC patients from The Cancer Genome Atlas (TCGA). A total of 5,564 lncRNAs and 15,767 mRNAs are expressed in > 80% of the tissue samples in the TCGA dataset. Due to differences between the platforms used in our study and the TCGA dataset, only commonly annotated lncRNAs and mRNAs in both studies were compared for differential expression and clinical association analysis. Among the commonly annotated lncRNAs and mRNAs in the two studies, 88% and 76% of the differentially expressed lncRNAs and mRNAs in our study are expressed in the same trend as the lncRNAs and mRNAs in TCGA HCC cohort, respectively. Notably, 53%, 76%, 81% and 86% lncRNAs that are significantly associated with Cancer Stage, Vascular invasion, Tumor grade and Overall survival in our study are expressed in the same trend in the same clinical group as the lncRNAs in TCGA dataset, while 67%, 67%, 95% and 90% mRNAs that are significantly associated with Cancer Stage, Vascular invasion, Tumor grade and Overall survival in our study are expressed in the same trend in the same clinical group as the mRNAs in TCGA dataset. Hence, the differential expression and clinical association of lncRNAs and mRNAs in this study is generally consistent with TCGA dataset.

## Discussion

HCC incidence has doubled from 2.6 per 100,000 populations to 5.2 per 100,000 populations in the past 20 years but the molecular mechanisms in HCC remained ambiguous^30,31^. Research on the role of lncRNAs in cancer has intensified as lncRNAs were found to regulate almost every phase of a gene’s life cycle^6^ and they are significantly de-regulated in several cancer types. Potential function of lncRNAs are often inferred through genes that either reside near (*in cis*) the lncRNAs or are co-expressed with the lncRNAs using the “guilt by association” strategy^15–23^ and pathways that they deregulate inferred through their co-expressed genes. This strategy was experimentally found to be useful to identify lncRNAs-mRNA pairs^32^.

Here, we present a novel approach to identify clinically important lncRNAs, associated with key clinical characteristics modulating the prognosis of HCC patients that have potential to serve as master regulators of genes in pertinent cancer pathways. Significantly co-expressed pairs of differentially expressed lncRNAs and mRNAs in the tumors of HCC patients were first identified using the “guilt by association” strategy. These lncRNAs and mRNAs were then further analyzed for their association with key clinical characteristics that modulate prognosis. Network analyses was then performed on the clinically relevant lncRNA-gene pairs and the cancer pathways that the co-expressed genes in the network reside were then inferred.

Although a total of 37 clinically-relevant networks can be generated (Figure S1), most comprise only one or a few lncRNAs that are co-expressed with only one or a few mRNAs. To identify the potential master oncogenic and tumour suppressor regulators, the network should preferably be in a pertinent cancer pathway and contain a few lncRNAs co-expressed with a large number of genes, where > 80% of the co-expressed genes have similar clinical significance as the correlated lncRNAs. Notably, we identified an oncogenic network comprising 5 potential oncogenic lncRNAs (G073851, PTTG3P, RACGAP1P, GSE61474_XLOC_040880 and CTD-2267D19.6) that may serve as possible master regulators as they are significantly co-expressed |R|≥ 0.90 with 91 genes enriched in the cell-cycle and Rho-GTPase signaling pathway. Cell cycle and Rho GTPases signaling pathways are frequently reported to be deregulated in HCC thus far^33–35^ but this is the first report that demonstrated the link between the lncRNAs, cell-cycle and Rho-GTPase signaling pathway and their association with tumor grade. Among the 5 potential oncogenic lncRNAs, both PTTG3P and RACGA1P were previously reported to be pseudogenes that were overexpressed in HCC and were also shown to enhance cell cycle progression and activate Rho-GTPase signaling pathways respectively^36–38^. The other 3 lncRNAs (G073851, GSE61474_XLOC_040880 and CTD-2267D19.6) are novel lncRNAs that were annotated by Iyer et al.^27^, Clark et al. ^28^ and GENCODE^39–41^, respectively and have not been studied previously in HCC.

When either of 2 potential oncogenic master regulators lncRNAs, GSE61474_XLOC_040880 (lncRNA-GSE) and CTD-2267D19.6 (lncRNA-CTD), were introduced into the immortalized LO2 cells, genes that were over-expressed were found to reside primarily in the transcription regulation pathways (Figs. 5b,c). RNA Pol I is responsible for transcription of rRNAs^42^, which is one of the limiting steps in cell cycle progression^43^, and its over-activation has been known as a hallmark for certain cancers^44^. Hence, it would be worthwhile to further investigate if both lncRNA-GSE and lncRNA-CTD activate RNA Pol I activity, leading to stimulation of rRNA synthesis and results in an increase in cell cycle progression. Genes involved in metabolism were found to be downregulated in cells that over-expressed either of these 2 lncRNAs (Figs. 5d,e). Intriguingly, proteoglycan deregulated by lncRNA-GSE such as decorin, was known to be a tumor suppresor^45–50^ and was reported to modulate cell cycle progression in HepG2 cells^51^. On the other hand, downregulation of fatty acid degradation by lncRNA-CTD could be due to the requirement of fatty acid for synthesis of membranes in rapidly proliferating cancer cells^52^. Given the close relationship between cell proliferation and metabolism^53^, we hypothesize that together these lncRNAs may inhibit genes in the major metabolic pathways to re-channel resources for cell proliferation.

Unlike the oncogenic network of lncRNA-gene, lncRNAs with potential to serve as master regulators are less obvious for the lncRNA-gene tumor suppressor networks. Though less obvious, the most promising tumor suppressor network comprise 4 lncRNAs (MT1DP isoforms (NR_027781 and NR_003658) and MT1IP isoforms (NR_003669 and NR_104046)) that are co-expressed with 8 genes in the metallothionein family (Fig. 6a,b). Similar pathways are also associated with lncRNAs that are specifically associated with tumor invasion (Fig. 7d). Lower expression of all the 4 lncRNAs and 7/8 of the co-expressed metallothionein family genes were found to be associated with tumor invasion (Fig. 6a–f) suggesting that this network may modulate metastasis. The co-expressed genes, MT1E and MT1H, were previously associated with tumour invasion in bladder, glioma, liver and prostate cancer respectively^44,54–57^. One of the lncRNAs, MT1IP was reported to act as a tumor suppressor in liver cancer by attenuating cell proliferation and transformation while inducing apoptosis^58^. However, none of the previous literature reported the correlation between the lncRNAs and metallothionein genes.

To address whether specific lncRNAs modulate specific clinical phenotype through unique pathways, lncRNAs that are only associated with a single clinical phenotype were identified and the pathways they deregulate were explored. Similar to the oncogenic network identified earlier (Fig. 4) which is primarily associated with tumor grade and capsule, up-regulated genes correlated with lncRNAs that are associated only with tumor grade reside in the cell-cycle and Rho GTPase signaling pathways (Fig. 7b, Table S2). On the other hand, down-regulated genes correlated with lncRNAs that are uniquely associated with tumor grade are enriched in glucose, fatty-acid (including arachidonic (AA)) and drug metabolism pathways (Fig. 7c) consistent with observations made when the oncogenic master-regulator, lncRNA-CTD was introduced into liver cells (Fig. 5e, Table S2). AA is a precursor of eicosanoids such as leukotrienes, which are inflammatory mediators and aberrant AA metabolism was previously reported in cancer^59,60^. Hence, tumor grade specific lncRNAs may modulate inflammation through AA metabolism in HCC, an inflammation-associated cancer ^61,62^. Genes correlated with lncRNAs that are uniquely associated with tumor grade in the glucose pathway that are down-regulated include ALDOB and ALDOC (Table S1a, boxed in orange) which are glycolytic adolase enzyme isoforms that cleaves Fructose 1,6-biphosphate to produce Dihydroxyacetone phosphate and Glyceraldehyde 3-phosphate^63^. ALDOB was reported to suppress metastasis in vitro and in vivo in HCC while ALDOC was reported with the same metastatic effect in oral squamous cell carcinoma^64,65^. It is thus worthwhile to further investigate the role of these tumor grade specific lncRNAs in modulating these 2 genes to affect metastatic potential.

Similarly, consistent with the tumor suppressive network identified earlier (Fig. 6), the 15 down-regulated genes that correlated with the 10 down-regulated lncRNAs associated with tumor invasion were metallothionein genes or genes involved in cellular response to external stimuli or metal ions (Table S1b and S2; Fig. 7d) highlighting the role of metallothionein family of genes in modulating tumor invasion and metastasis. No lncRNAs or correlated genes were found to be upregulated in invasive tumors. Only a single up-regulated gene, DCAF13 (DDB1 and CUL4 associated factor 13) (Table S1c, boxed in orange) was found to be correlated with the only up-regulated tumor capsule specific lncRNA, DCAF13P3 (Table S1c, boxed in orange). Consistent with our observations, DCAF13 was also previously reported to be upregulated in HCC and correlated with MYC mRNA and protein level^66^, although its correlation with the DCAF13P3 lncRNA or its association with tumor capsulation in HCC had not been previously reported. Nonetheless, 9 down-regulated genes which are correlated with 8 down-regulated tumor capsule specific lncRNAs, were found to be mainly involved in serine hydrolase and serine-type peptidase activity (Fig. 7e). Interestingly, serine type peptidase such as KLK2 (Table S1c, boxed in orange) are reported to activate plasminogen activator which plays important role in extracellular matrix (ECM) degradation^67–69^ suggesting that these lncRNAs may down-regulate serine-type peptidase so that tumor capsule can remain intact.

Although no lncRNAs were up-regulated, 4 lncRNAs (LINC01554, G019663, XLOC_006182 and XLOC_007985) were found to be down-regulated in patients with worse overall survival (Table S1d). These 4 lncRNAs were correlated with 7 genes (RUFY4, C5orf27, KRTAP4-1, KRTAP10-8, AK055785, KLK2 and SERPINB12) which are also down-regulated in the tumors of patients. Decreased expression of two of these genes, C5orf27 and KRTAP4-1 were associated with poorer overall survival. Functions of most of these genes remain unknown. Hence, it may be worthwhile to further characterize the role of these lncRNAs and their correlated novel genes for their role in modulating patient overall survival.

Two down-regulated tumor-property-specific lncRNAs (XLOC_010739, G015949) was found to be correlated with 4 down-regulated genes (PRRT3, AL590560.1, AC114783.1 and AZGP1) (Table S1e, boxed in orange). Only the AZGP1 gene has previously been reported to be down-regulated in the tumors of HCC patients and associated with poorer prognosis^70,71^. The down-regulation of AZGP1 was reported to be associated with histone acetylation and thought to promote tumor progression through PTEN/Akt and CD44s pathways^71^.

Although these findings have provided novel insights, some limitations still remain. In this study, microarray was employed to quantify the expression levels of lncRNAs, rather than RNA sequencing, which may hinder the discovery of novel lncRNAs. RNA sequencing was not used in this study as lncRNAs are known to be expressed at low levels in the cells and low abundant transcripts are not reliably quantified by RNA sequencing^72,73^. An increase in read depth was shown to have no impact on the expression values of poorly expressed transcripts but only increase the expression values of abundantly expressed transcripts^74^. Additionally, Labaj, et al., demonstrated that 75% of the sequencing reads concentrated on only 7% of the highly abundant transcript^73^, thereby hindering the precise detection of low abundant lncRNA transcripts.

**Figure 8:**
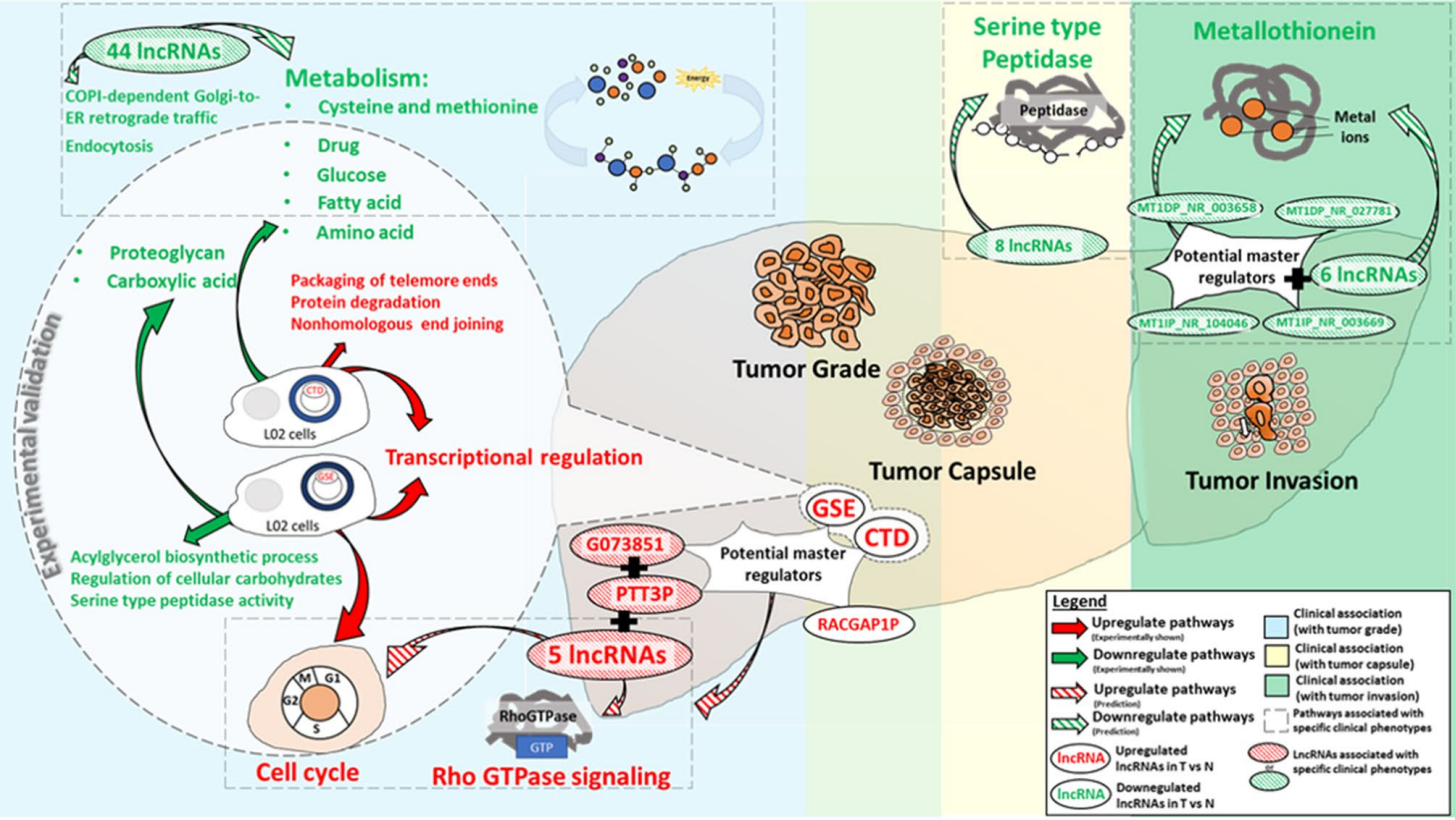
Summary of the key lncRNAs associated with prognosis of HCC patients and the pathways that they modulate.

In conclusion, this study which identifies key lncRNA signatures associated with key clinical phenotype, which is presented in Fig. 8, provides valuable insights into the important role of lncRNAs in modulating prognosis of patients. The identification of potential oncogenic and tumor suppressive networks of lncRNAs-genes facilitates design of therapy that target these key lncRNA signature in pertinent cancer networks. It may thus be worthwhile to further investigate and experimentally validate both the oncogenic and tumor suppressor networks of lncRNA-genes. If validated, this strategy may be employed to facilitate the identification of key targetable master regulator of genes that play roles in modulating prognosis of not only HCC, but other cancers as well.

## Materials and methods

### Preparation of patient tissues samples

Tumors tissues and adjacent non-tumorous tissues were obtained from forty-nine patients who underwent surgery at the Singapore General Hospital. Informed consent was obtained from all forty-nine patients. The tissues were frozen immediately with liquid nitrogen after collection from surgery and were stored in liquid nitrogen until use. All the tissues were collected anonymously with informed consent from the patients and prior approval from the SingHealth Institutional Review Board (SingHealth CIRB Ref: 2018/3155). All methods were carried out in accordance with relevant guidelines and regulations.

### RNA extraction, labeling and hybridization on array

Frozen tissue samples were homogenized by gentleMACS dissociator (Miltenyi Biotec) in RLT buffer (Qiagen, Germany) with 1% β-mercaptoethanol. Total RNA was then extracted from tumor tissues and adjacent non-tumorous tissues using RNeasy mini kit (Qiagen, Germany) following the manufacturer’s protocol. The extracted RNA was quantified using NanoDrop ND-1000 spectrometer (Nanodrop Products, USA). RNA integrity was checked using Agilent 2,100 Bioanalyzer. Samples preparation and microarray hybridization were carried out following Agilent array protocols with minor modifications. The RNA samples were amplified and transcribed into fluorescent cRNA without 3′ bias using a random priming method (Arraystar Flash RNA Labeling Kit, Arraystar), followed by hybridization onto Human LncRNA Array v4.0 (8 × 60 k, Arraystar). The array was then washed and scanned with Agilent DNA Microarray Scanner G2505C.

### LncRNA and mRNA microarray data analysis

The array images were analyzed using Agilent Feature Extraction software (version 11.0.1.1). Subsequently, quantile normalization and data processing were performed by GeneSpring GX v12.1 software package (Agilent Technologies). LncRNA and mRNA that have flags in Present or Marginal in at least 50% samples were used for data analysis. To identify differentially expressed lncRNAs/mRNAs, Partek Genomics Suite (Partek lnc., USA) was used to perform paired t-tests on the normalized intensity of each lncRNAs/mRNAs and filter for absolute fold change ≥ 2 and false discovery rate (FDR) adjusted *P* value < 0.05. Hierarchical clustering was also carried out on the significantly deregulated lncRNAs/mRNAs.

### Association of lncRNAs with pertinent clinical characteristics

The 8 different clinical characteristics were grouped into 5 categories, namely, ‘tumor properties’ (tumor size, vascular invasion and tumor stage), tumor grade (or Edmonson grade), tumor capsule (encapsulation and degree of encapsulation), tumor invasion and overall survival status (Table S3). For each clinical phenotype, the tumor samples were classified as either ‘good characteristics’ (tumor size < 5 cm; no vascular invasion, stage 1/2, grade 1/2, complete encapsulation, no tumor invasion) or ‘poor characteristics’ (tumor size ≥ 5 cm; vascular invasion, stage 3/4, grade 3/4, no or incomplete encapsulation, tumor invasion) (Table S3). Normalized intensity for each lncRNAs/mRNAs between the poor clinical characteristics and good clinical characteristics for tumor properties, tumor grade, tumor capsule and tumor invasion were then analyzed with Student t test using Partek Genomics Suite (Partek lnc., USA). Absolute fold change ≥ 1.5 and unadjusted *P* value < 0.05 are considered as significant. For overall survival analysis, univariate cox regression was applied using Partek Genomics Suite (Partek lnc., USA). LncRNAs with hazard ratio (HR) > 1 or < 1 with unadjusted *P* value < 0.05 are considered as significant. LncRNAs are considered as potentially oncogenic if higher tumor expression of the lncRNA is associated with clinical characteristics, including larger tumor size, higher tumor grade, non-encapsulated tumor, invasive tumor, poorer overall survival (HR > 1) that augur poorer prognosis. On the other hand, lncRNAs are considered potential tumor suppressive if lower tumor expression of the lncRNA is associated with clinical features that characterize poorer prognosis.

### Clinically associated co-expression networks constructions

To identify lncRNAs – mRNA co-expressing pairs, a Pearson correlation coefficient (PCC) was calculated based on normalized intensity between all differentially expressed lncRNAs and mRNAs using miRComb R package^75^. A PCC value ≥ 0.9 was considered as strong correlation. LncRNAs-mRNA co-expressing pairs were then filtered to only those containing clinically relevant lncRNAs for network analysis. The clinically associated co-expression networks were drawn using the Cytoscape software ^26^.

### Gene ontology and pathway analysis

Gene ontology and pathway analysis were performed using ConsensusPathDB^24,25^ based on the Kyoto Encyclopedia of Genes and Genomes (KEGG)^76^ and Reactome pathways^77^ databases. The enriched pathways with a value of *P* < 0.01 are considered significant.

### Processing of TCGA RNA sequencing data

RNA sequencing data of 361 tumor tissues and 49 adjacent non-tumorous tissues of HCC patients was downloaded from TCGA (https://www.cancer.gov/tcga.) to obtain lncRNAs and mRNAs expression profile for analyses. Clinical data (Cancer Stage, Vascular invasion, tumor grade and overall survival) of patients was also retrieved from the TCGA website. Paired Student T-test was performed on 49 HCC paired tissue samples in the TCGA dataset to obtain differential expression of lncRNAs and mRNAs. Logistic regression was performed on cancer stage, vascular invasion and tumor grade while cox regression was used for overall survival analysis.

### Plasmid construction and preparation

The complete sequence of lncRNA-GSE cDNA was synthesized by Bio Basic Inc. and cloned downstream of human cytomegalovirus (CMV) promoter into pcDNA3.1 + plasmid (Thermo Scientific, USA) using *Nhe*1 and *Not*1. The complete RNA transcript of lncRNA-CTD was obtained from HepG2 cells as described: Total RNA was extracted using RNeasy Mini Kit (Qiagen), following manufacturer’s protocol. One microgram of RNA was treated with RQ1 RNase-free DNase (Promega, USA). Reverse transcription (RT) of RNA into cDNA was then performed using SuperScript™ II Reverse transcriptase (Invitrogen, USA) and random primers (Invitrogen, USA), according to manufacturer’s instructions. The lncRNA-CTD cDNA was then amplified using Expand High Fidelity PCR system (Roche, Switzerland), according to manufacturer’s protocol. The primer sequences used for amplification is shown in Table S4. lncRNA-CTD cDNA sequences were then cloned downstream of CMV promoter into pcDNA3.1 + plasmid (Thermo Scientific, USA) using *EcoR*1 and *Not*1. The plasmids were sequenced to ensure the sequences are correct without mutation before propagation. Maxi-preparation of plasmids was carried out using NucleoBond® Xtra Maxi EF kit (Macherey–Nagel, Germany), following manufacturer’s instructions. Both lncRNA-GSE and lncRNA-CTD cDNA sequences are shown in Table S5.

### Cell cultures and transfection

LO2, an immortalized liver cell-line was maintained in Dulbecco’s Modified Eagles’ medium (DMEM, Sigma Aldrich, USA) with 10% Fetal bovine serum (FBS, Biological Industries, Israel) and incubated in a 37 °C incubator with 5% CO_2_. These cells were kindly provided by Professor Guan Xin Yuan, Director of Laboratory of Cancer Genetics, Hong Kong University. To overexpress lncRNA-GSE and lncRNA-CTD in L02 cells, 50,000 cells were seeded on 6-well culture dish a day before transfecting pcDNA3.1 + containing lncRNA sequences using Dharmafect kb transfection reagent, following manufacturer’s protocol. pcDNA3.1 + plasmid (Thermo Scientific, USA) was used as a control in this experiment. Overexpression of lncRNAs was confirmed using multiplex PCR and agarose gel electrophoresis (Figure S3 and S4).

### Polymerase Chain reaction (PCR) and agarose gel electrophoresis

The successful overexpression of lncRNA targets in L02 cells (Figure S3 and S4) was validated through reverse transcription of extracted RNA and cDNA amplification using HotStarTaq® DNA polymerase according to manufacturer’s protocol. Primers used to amplify cDNA sequences of lncRNA-GSE and lncRNA-CTD are shown in Table S4. PCR products were separated on a 1% (w/v) agarose gel.

### Real time RT-PCR

Expression of lncRNA targets in patient samples (Figure S2) was determined through real-time RT-PCR using SYBR™ Green PCR master mix (Life Technologies, UK) on the Applied Biosystem™ 7,500 Real-Time PCR systems (Applied Biosystems, USA), following manufacturer’s protocol. Primers used to amplify cDNA sequences of lncRNA-GSE and lncRNA-CTD in real time RT-PCR are shown in Table S4. All expression was normalized to actin and 2-^ΔΔCt^ was used to calculate relative expression of each gene.

### RNA sequencing of LO2 cells transfected with GSE/CTD lncRNAs

Total RNA from LO2 cells transfected with GSE/CTD lncRNAs was extracted using RNeasy Mini Kit (Qiagen) according to manufacturer’s protocol. The quality of RNA was evaluated using Nanodrop, Agarose gel electrophoresis and Agilent 2,100. mRNA was first purified using poly-T-oligo-attached magnetic beads, followed by random fragmentation. Random hexamers and M-MuLV reverse transcriptase were used to synthesize first strand cDNA and subsequently DNA Polymerase I and RNase H were used to synthesize second strand cDNA. Then, sequencing adaptors was ligated to the cDNAs after converting overhangs into blunt ends and adenylation of 3′ends. Fragments of 150-200 bp long were selected via purification using AMPure XP system (Beckman Coulter, Beverly, USA), followed by PCR amplification and final purification of PCR products using AMPure XP beads. Libraries were then fed into illumina machines for sequencing after quality assessment using Qubit2.0, Agilent 2,100 and Q-PCR. Next, raw image data file was transformed to Sequencing Reads using CASAVA base recognition (Base Calling). Raw reads were filtered to remove low quality reads or reads containing adaptors, followed by mapping the clean reads to human reference genome using STAR software. Subsequently, readcount of the genes was adjusted by TMM and differential analysis was performed by EdgeR R package.

## Supplementary information

Supplementary file1 (PDF 3756 kb)

## Data Availability

The microarray data generated in this study is available in Gene Expression Omnibus with series entry GSE138178.
