# Supplementary material for: Network of clinically-relevant lncRNAs-mRNAs associated with prognosis of hepatocellular carcinoma patients
Source: Sci Rep. 2020 Jul 7;10:11124. doi: 10.1038/s41598-020-67742-8 (PMC7341759; doi:10.1038/s41598-020-67742-8)
Supplement: Supplementary file 1 — Supplementary file1 (PDF 3756 kb) [file 41598_2020_67742_MOESM1_ESM.pdf]

Supplemental Information

Network of clinically-relevant lncRNAs-mRNAs associated with prognosis of Hepatocellular Carcinoma patients

Lee Jin Lim[1], Yu Jin[2,3], Henry Yang[4], Alexander Y.F. Chung[5], Brian K.P. Goh[5], Pierce K.H. Chow[5,6,7] , Chung Yip Chan[5], William K. Blanks[1,8], Peng Chung Cheow[5], Ser Yee Lee[5], Tony K.H. Lim[9], Samuel S. Chong[10], London L.P.J.Ooi[5,6,7] and Caroline G.Lee[1,2,3,6*]

*Corresponding author's email: bchleec@nus.edu.sg

This file includes:

Table of contents

Figure S1 2
Figure S2 3
Figure S3 4
Figure S4 5
Table S1a 6
Table S1b 7
Table S1c 8
Table S1d 9
Table S1e 10
Table S2 11
Table S3 12
Table S4 13
Table S5 14

Figure S1

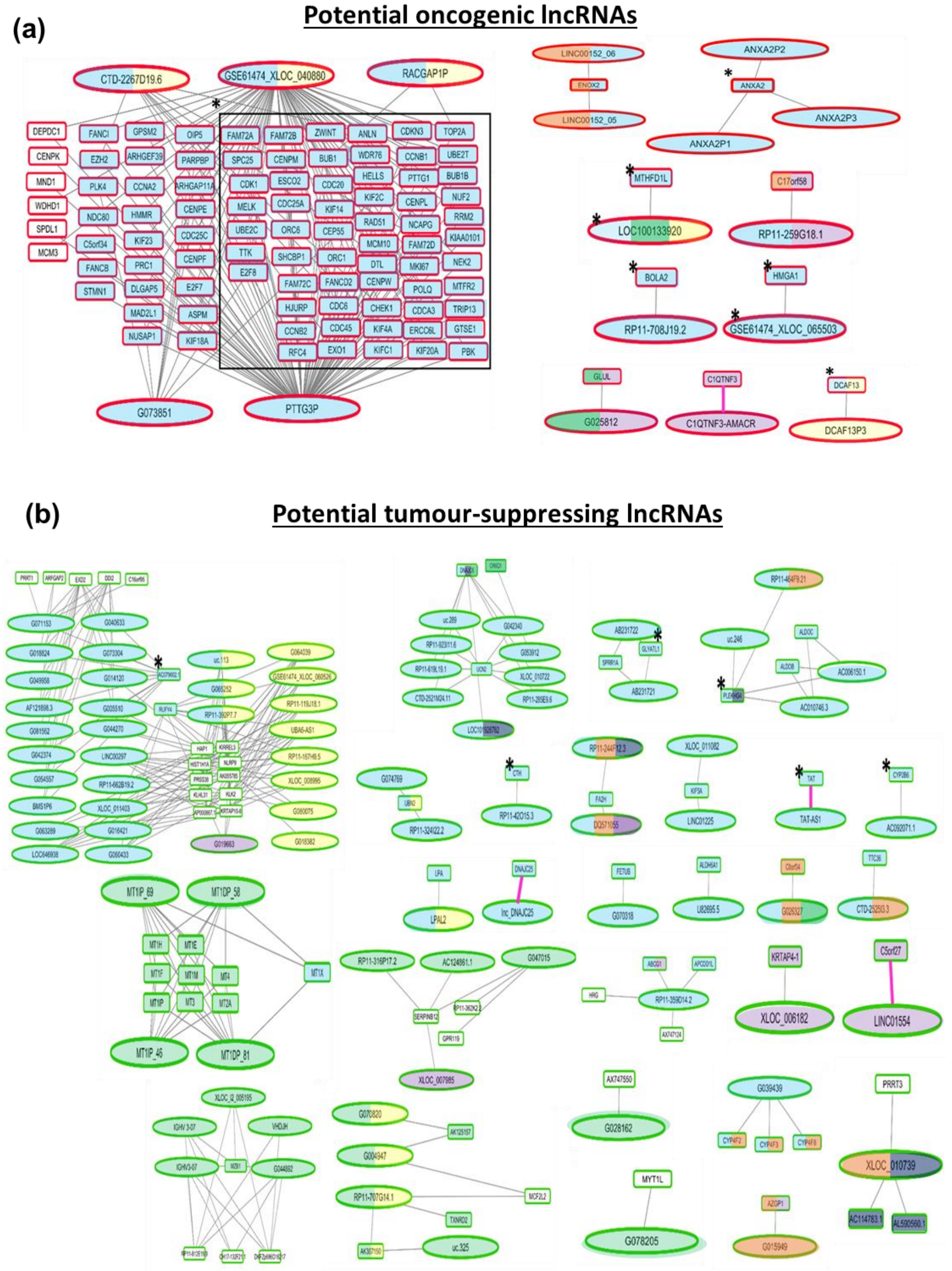

Figure S1 Networks containing potentially oncogenic (top) or tumor suppressing (bottom) lncRNAs. Oval shape: lncRNAs; Square: mRNAs; Black lines: Strong correlation. Outline of oval and square represents lncRNAs/mRNAs expression in T vs NT; Red: Upregulation, Green: Downregulation. Colours within oval/square represents the clinical significance of lncRNAs/mRNAs. Blue background: Edmondson Grade; Orange background: Tumor properties (Includes tumor size, tumor stage and vascularization); Purple background: Overall survival; Green background: Tumor invasion; Yellow background: Tumor capsule (Includes Encapsulation and Degree of encapsulation); *: clinical significance with FDR<0.05. The network was drawn using Cytoscape software (Version 3.5.1) (http://cytoscape.org).

Figure S2

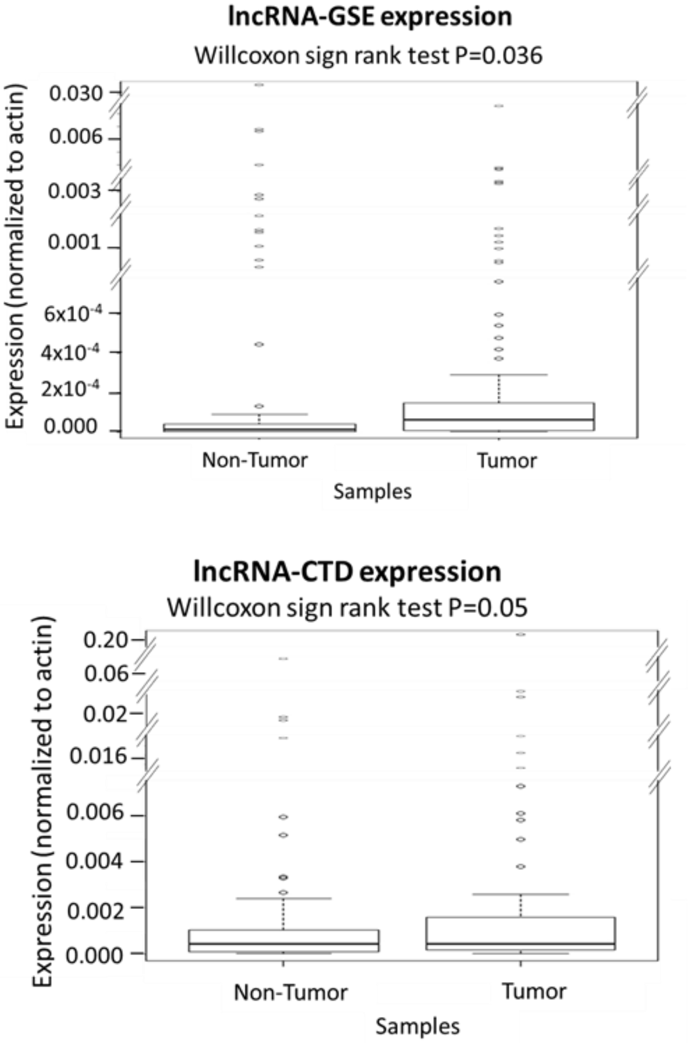

Figure S2 Validation of lncRNA-GSE (Top panel) and lncRNA-CTD (bottom panel) using real time qPCR. The box plots shows the expression of lncRNA-GSE (Top) /lncRNA-CTD (bottom) in non-tumor and tumor tissues in 59 patients.

Figure S3

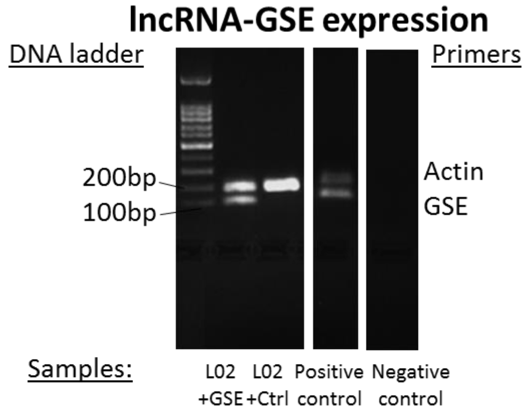

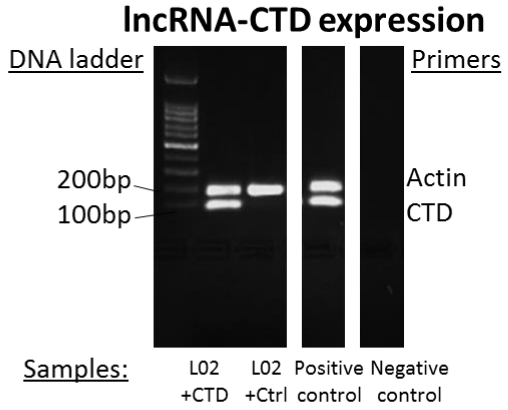

Figure S3 Figures shows the multiplex PCR result of lncRNA-GSE (Top panel) and lncRNA-CTD (bottom panel) expression after transfection. L02+GSE: L02 cells transfected with pcDNA3.1+containing lncRNA-GSE sequences. L02+Ctrl: L02 cells transfected with pcDNA3.1+ plasmid. L02+CTD: L02 cells transfected with pcDNA3.1+containing lncRNA-CTD sequences. The images are cropped from different parts of the same gel. Full-length gel image is presented in Figure S4.

Figure S4

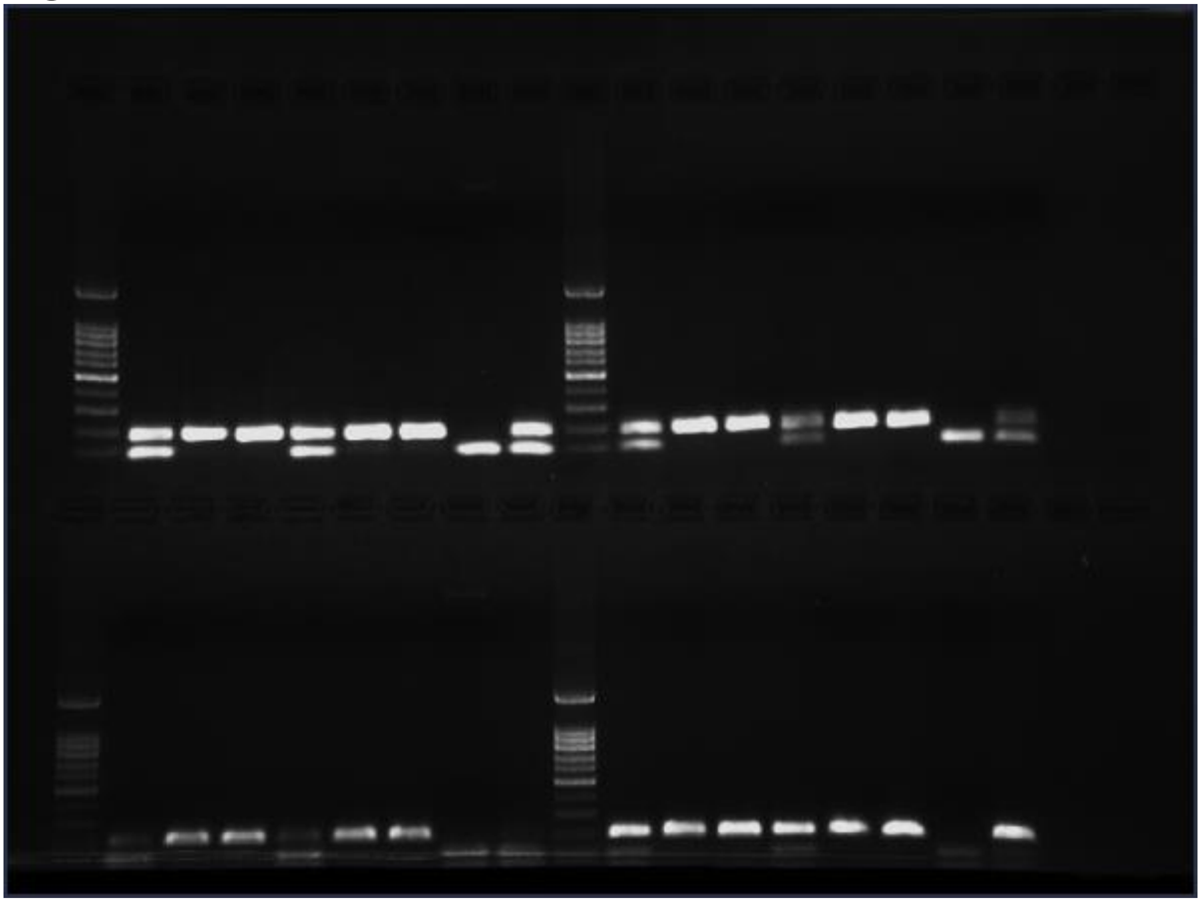

Figure S4: Full-length gel image of Figure S3. Multiplex PCR result of lncRNA-GSE and lncRNA-CTD expression after transfection are located at the top right panel and top left panel respectively.

TableS1a: Correlation between tumor grade specific lncRNAs and mRNAs

Tumor Grade		Clinical association	TG	TG	TG	TG	TG	TG	TG	TG	TG	TG	TG	TG	TG	TG	TG	TG	TG	TG	TG	TG	TG	TG	TG	TG	TG	TG	TG	TG	TG	TG	TG	TG	TG	TG	TG	TG	TG	TG	TG	TG	TG	TG	TG	TG	TG	TG	TG	TG	TG	TG	TG
		FC	2.44	2.36	2.23	2.13	8.46	5.37	2.79	-3.55	-4.24	-4.24	-4.61	-4.74	-5.75	-6.30	-6.88	-7.11	-3.79	-4.06	-4.37	-6.25	-3.31	-3.47	-4.22	-4.47	-2.89	-2.90	-3.49	-3.63	-3.65	-2.98	-4.35	-2.58	-3.34	-3.43	-2.75	-4.89	-2.09	-5.33	-2.67	-14.83	-3.14	-2.83	-3.10	-2.41	-3.79	-4.08	-3.80	-2.13	-2.09	-2.11	-3.19
Clinical association	FC	Gene Symbol	GSE61474_XLOC_065503	RP11-708J19.2	ANXA2P2	ANXA2P3	PTTG3P	G073851	ANXA2P1	G018824	LOC646938	G005510	G081562	RP11-662B19.2	G073304	G040633	LINC00297	G044270	G053912	G071153	BMS1P6	G054557	RP11-285E9.6	CTD-2521M24.11	G014120	RP11-324I22.2	G016421	G049958	uc.246	uc.289	AB231722	G042374	XLOC_010722	G042340	G074769	AC006150.1	RP11-619L19.1	XLOC_011403	DNAJC25	AB231721	U82695.5	AC092071.1	G039439	LINC01225	G063289	RP11-923I11.6	RP11-42O15.3	AC010746.3	TAT-AS1	XLOC_011082	G070318	RP11-359D14.2	AF121898.3
	-3.4	EXD2	-0.44	-0.59	-0.57	-0.50	-0.65	-0.51	-0.52	0.92	0.84	0.94	0.90	0.89	0.97	0.96	0.76	0.89	0.59	0.98	0.95	0.97	0.57	0.58	0.94	0.58	0.78	0.93	0.59	0.63	0.54	0.94	0.56	0.53	0.61	0.57	0.53	0.79	0.54	0.51	0.55	0.57	0.45	0.41	0.84	0.54	0.62	0.53	0.54	0.41	0.49	0.38	0.93
TG	-2.9	AC079602.1	-0.54	-0.66	-0.63	-0.58	-0.69	-0.59	-0.60	0.92	0.84	0.91	0.89	0.85	0.96	0.94	0.77	0.89	0.60	0.91	0.96	0.94	0.59	0.61	0.91	0.62	0.75	0.91	0.62	0.59	0.60	0.92	0.57	0.57	0.62	0.63	0.54	0.75	0.60	0.57	0.58	0.63	0.49	0.50	0.84	0.57	0.65	0.58	0.58	0.46	0.52	0.44	0.93
	-3.2	DDI2	-0.48	-0.61	-0.60	-0.53	-0.69	-0.53	-0.56	0.92	0.84	0.91	0.89	0.86	0.95	0.94	0.74	0.88	0.60	0.97	0.91	0.94	0.57	0.59	0.91	0.61	0.78	0.91	0.62	0.64	0.57	0.91	0.56	0.55	0.61	0.62	0.55	0.76	0.57	0.55	0.56	0.62	0.45	0.44	0.83	0.56	0.64	0.57	0.58	0.43	0.51	0.42	0.91
TG	-3.3	UCN2	-0.75	-0.61	-0.61	-0.55	-0.74	-0.59	-0.63	0.63	0.57	0.59	0.64	0.58	0.62	0.65	0.61	0.61	0.90	0.58	0.62	0.64	0.98	0.95	0.59	0.76	0.44	0.50	0.67	0.92	0.73	0.58	0.91	0.98	0.76	0.63	0.97	0.51	0.62	0.73	0.80	0.54	0.72	0.78	0.53	0.90	0.66	0.64	0.63	0.77	0.57	0.53	0.59
	-2.7	HAP1	-0.39	-0.64	-0.57	-0.51	-0.67	-0.55	-0.56	0.82	0.93	0.93	0.89	0.93	0.90	0.83	0.86	0.92	0.59	0.86	0.84	0.89	0.53	0.54	0.92	0.52	0.90	0.84	0.58	0.56	0.50	0.84	0.51	0.51	0.52	0.56	0.49	0.87	0.53	0.48	0.52	0.52	0.36	0.46	0.93	0.49	0.54	0.50	0.48	0.42	0.41	0.27	0.88
TP TG TI	-4.8	DNAJC6	-0.75	-0.71	-0.61	-0.55	-0.77	-0.63	-0.63	0.70	0.66	0.69	0.71	0.67	0.70	0.70	0.68	0.70	0.89	0.63	0.69	0.71	0.94	0.93	0.67	0.75	0.54	0.60	0.68	0.91	0.71	0.66	0.94	0.93	0.76	0.64	0.93	0.58	0.67	0.71	0.82	0.59	0.69	0.75	0.64	0.83	0.70	0.62	0.65	0.73	0.57	0.46	0.71
	-3.1	KRTAP10-8	-0.46	-0.68	-0.63	-0.59	-0.69	-0.66	-0.63	0.68	0.95	0.83	0.79	0.90	0.76	0.63	0.90	0.87	0.54	0.67	0.70	0.74	0.47	0.48	0.86	0.49	0.96	0.65	0.55	0.42	0.48	0.64	0.37	0.45	0.44	0.56	0.42	0.89	0.53	0.47	0.43	0.55	0.38	0.49	0.95	0.43	0.49	0.49	0.40	0.34	0.46	0.30	0.75
	-4.2	NLRP9	-0.52	-0.71	-0.61	-0.57	-0.73	-0.69	-0.60	0.75	0.96	0.87	0.87	0.94	0.82	0.73	0.95	0.92	0.61	0.72	0.80	0.79	0.57	0.58	0.86	0.60	0.88	0.74	0.62	0.51	0.56	0.76	0.50	0.55	0.56	0.60	0.49	0.87	0.59	0.54	0.54	0.62	0.45	0.56	0.93	0.49	0.59	0.53	0.50	0.45	0.46	0.31	0.81
	-4.5	AK055785	-0.46	-0.71	-0.63	-0.59	-0.69	-0.65	-0.62	0.66	0.92	0.80	0.81	0.89	0.73	0.61	0.91	0.87	0.58	0.68	0.68	0.72	0.49	0.49	0.84	0.53	0.92	0.62	0.54	0.44	0.52	0.64	0.38	0.47	0.47	0.56	0.43	0.92	0.50	0.51	0.43	0.53	0.41	0.51	0.89	0.48	0.49	0.49	0.41	0.39	0.47	0.33	0.70
TG	-4.6	KLK2	-0.50	-0.67	-0.63	-0.58	-0.72	-0.66	-0.61	0.66	0.93	0.82	0.83	0.91	0.75	0.65	0.95	0.89	0.64	0.69	0.70	0.74	0.57	0.58	0.84	0.59	0.89	0.63	0.57	0.52	0.55	0.67	0.48	0.55	0.55	0.56	0.51	0.92	0.56	0.54	0.51	0.52	0.43	0.56	0.88	0.53	0.51	0.49	0.44	0.43	0.43	0.31	0.71
TG	2.4	ANXA2	0.69	0.65	0.97	0.96	0.68	0.59	0.98	-0.64	-0.64	-0.57	-0.61	-0.61	-0.56	-0.52	-0.63	-0.57	-0.51	-0.52	-0.58	-0.57	-0.58	-0.54	-0.60	-0.71	-0.62	-0.53	-0.64	-0.46	-0.65	-0.52	-0.49	-0.55	-0.67	-0.69	-0.55	-0.59	-0.70	-0.63	-0.65	-0.54	-0.61	-0.56	-0.66	-0.60	-0.66	-0.64	-0.47	-0.51	-0.61	-0.54	-0.61
TP TG	-3.0	PLEKHG4	-0.71	-0.60	-0.63	-0.59	-0.66	-0.57	-0.61	0.66	0.60	0.60	0.58	0.57	0.64	0.62	0.56	0.59	0.54	0.53	0.66	0.65	0.59	0.53	0.61	0.65	0.50	0.58	0.95	0.49	0.77	0.62	0.55	0.59	0.61	0.92	0.55	0.50	0.69	0.73	0.67	0.69	0.62	0.57	0.61	0.63	0.78	0.93	0.75	0.56	0.64	0.65	0.67
	-3.0	KIRREL3	-0.47	-0.63	-0.64	-0.59	-0.68	-0.60	-0.61	0.71	0.91	0.85	0.88	0.91	0.79	0.72	0.93	0.90	0.62	0.73	0.77	0.78	0.60	0.59	0.85	0.61	0.83	0.71	0.58	0.55	0.55	0.76	0.52	0.57	0.60	0.55	0.51	0.88	0.58	0.52	0.53	0.47	0.44	0.55	0.85	0.53	0.54	0.48	0.43	0.47	0.40	0.28	0.74
TG	18.4	E2F8	0.71	0.78	0.67	0.63	0.96	0.90	0.68	-0.69	-0.78	-0.69	-0.68	-0.73	-0.69	-0.67	-0.77	-0.74	-0.68	-0.61	-0.67	-0.67	-0.69	-0.69	-0.68	-0.69	-0.64	-0.58	-0.72	-0.59	-0.71	-0.62	-0.61	-0.70	-0.64	-0.71	-0.69	-0.65	-0.67	-0.69	-0.65	-0.70	-0.65	-0.68	-0.76	-0.62	-0.64	-0.66	-0.60	-0.56	-0.60	-0.48	-0.69
TG	13.0	CDK1	0.76	0.80	0.71	0.68	0.97	0.90	0.72	-0.72	-0.75	-0.66	-0.66	-0.70	-0.67	-0.65	-0.73	-0.70	-0.67	-0.59	-0.67	-0.65	-0.70	-0.69	-0.65	-0.75	-0.61	-0.58	-0.75	-0.59	-0.76	-0.61	-0.62	-0.71	-0.71	-0.74	-0.70	-0.61	-0.72	-0.74	-0.70	-0.75	-0.71	-0.67	-0.74	-0.64	-0.70	-0.71	-0.62	-0.58	-0.67	-0.57	-0.70
TG	9.2	OIP5	0.71	0.78	0.66	0.62	0.95	0.91	0.67	-0.70	-0.76	-0.67	-0.67	-0.72	-0.68	-0.64	-0.76	-0.71	-0.71	-0.61	-0.66	-0.65	-0.72	-0.71	-0.67	-0.71	-0.63	-0.55	-0.69	-0.64	-0.69	-0.60	-0.63	-0.71	-0.67	-0.67	-0.71	-0.65	-0.66	-0.69	-0.68	-0.72	-0.70	-0.65	-0.73	-0.64	-0.66	-0.63	-0.59	-0.55	-0.61	-0.50	-0.69
TG	9.0	CCNB1	0.77	0.81	0.69	0.65	0.97	0.91	0.71	-0.74	-0.77	-0.69	-0.68	-0.73	-0.70	-0.68	-0.76	-0.73	-0.70	-0.62	-0.68	-0.68	-0.72	-0.72	-0.68	-0.75	-0.63	-0.60	-0.76	-0.64	-0.77	-0.63	-0.65	-0.72	-0.70	-0.76	-0.71	-0.63	-0.72	-0.75	-0.71	-0.74	-0.72	-0.68	-0.75	-0.65	-0.72	-0.72	-0.64	-0.60	-0.67	-0.56	-0.73
TG	7.6	PARPBP	0.70	0.77	0.65	0.60	0.93	0.91	0.69	-0.71	-0.79	-0.69	-0.67	-0.74	-0.68	-0.64	-0.78	-0.73	-0.67	-0.60	-0.67	-0.66	-0.69	-0.68	-0.67	-0.70	-0.65	-0.59	-0.72	-0.58	-0.72	-0.60	-0.60	-0.69	-0.66	-0.72	-0.67	-0.66	-0.66	-0.71	-0.70	-0.75	-0.69	-0.67	-0.77	-0.60	-0.66	-0.69	-0.64	-0.57	-0.64	-0.53	-0.71
TG	7.4	ARHGAP11A	0.72	0.75	0.67	0.64	0.94	0.90	0.68	-0.69	-0.73	-0.65	-0.65	-0.69	-0.65	-0.63	-0.73	-0.68	-0.62	-0.57	-0.64	-0.63	-0.67	-0.65	-0.63	-0.73	-0.58	-0.55	-0.71	-0.56	-0.71	-0.59	-0.58	-0.67	-0.68	-0.70	-0.65	-0.58	-0.67	-0.71	-0.64	-0.72	-0.70	-0.66	-0.71	-0.59	-0.67	-0.65	-0.58	-0.55	-0.61	-0.52	-0.66
TG	7.2	CENPE	0.73	0.77	0.66	0.63	0.96	0.91	0.67	-0.66	-0.71	-0.62	-0.62	-0.67	-0.64	-0.62	-0.72	-0.67	-0.63	-0.54	-0.61	-0.61	-0.67	-0.65	-0.61	-0.71	-0.56	-0.51	-0.72	-0.55	-0.75	-0.58	-0.59	-0.68	-0.66	-0.70	-0.65	-0.58	-0.71	-0.73	-0.67	-0.71	-0.70	-0.65	-0.69	-0.59	-0.67	-0.67	-0.61	-0.54	-0.63	-0.52	-0.65
TG	5.7	MAD2L1	0.71	0.78	0.65	0.62	0.94	0.95	0.67	-0.67	-0.75	-0.64	-0.63	-0.69	-0.64	-0.60	-0.75	-0.68	-0.65	-0.55	-0.63	-0.61	-0.65	-0.66	-0.64	-0.65	-0.63	-0.52	-0.70	-0.55	-0.70	-0.55	-0.55	-0.66	-0.59	-0.71	-0.65	-0.63	-0.64	-0.71	-0.64	-0.73	-0.71	-0.67	-0.73	-0.60	-0.65	-0.67	-0.60	-0.56	-0.65	-0.56	-0.67
	3.7	PLK4	0.73	0.74	0.67	0.63	0.93	0.92	0.66	-0.67	-0.70	-0.62	-0.64	-0.67	-0.63	-0.61	-0.72	-0.66	-0.64	-0.54	-0.63	-0.61	-0.70	-0.69	-0.62	-0.71	-0.56	-0.51	-0.70	-0.58	-0.72	-0.56	-0.59	-0.70	-0.66	-0.70	-0.66	-0.57	-0.63	-0.72	-0.65	-0.72	-0.73	-0.69	-0.67	-0.62	-0.69	-0.67	-0.63	-0.59	-0.66	-0.57	-0.64
	-2.2	ARFGAP2	-0.58	-0.63	-0.63	-0.57	-0.70	-0.64	-0.64	0.96	0.81	0.84	0.79	0.79	0.88	0.86	0.69	0.79	0.58	0.86	0.85	0.87	0.53	0.57	0.84	0.61	0.73	0.83	0.61	0.56	0.68	0.78	0.51	0.53	0.58	0.70	0.51	0.66	0.61	0.67	0.57	0.70	0.58	0.50	0.82	0.55	0.71	0.64	0.65	0.43	0.59	0.58	0.90
TG	-2.5	KIF5A	-0.77	-0.66	-0.56	-0.52	-0.67	-0.65	-0.64	0.61	0.59	0.55	0.58	0.55	0.56	0.55	0.61	0.58	0.80	0.47	0.58	0.56	0.80	0.80	0.54	0.66	0.46	0.48	0.67	0.73	0.79	0.50	0.78	0.82	0.60	0.69	0.78	0.47	0.66	0.80	0.74	0.60	0.74	0.95	0.56	0.81	0.71	0.68	0.71	0.92	0.60	0.57	0.58
	-3.1	PRSS38	-0.50	-0.68	-0.59	-0.53	-0.75	-0.65	-0.57	0.71	0.90	0.84	0.85	0.91	0.78	0.71	0.93	0.88	0.65	0.73	0.73	0.77	0.62	0.61	0.83	0.62	0.82	0.68	0.54	0.59	0.55	0.72	0.55	0.59	0.61	0.50	0.55	0.86	0.57	0.53	0.59	0.51	0.47	0.57	0.84	0.54	0.52	0.45	0.46	0.51	0.45	0.28	0.75
TG TC	-3.1	UBN2	-0.72	-0.64	-0.74	-0.71	-0.69	-0.49	-0.63	0.69	0.56	0.62	0.67	0.61	0.64	0.67	0.62	0.63	0.62	0.57	0.65	0.65	0.74	0.68	0.61	0.96	0.42	0.55	0.64	0.69	0.69	0.64	0.69	0.70	0.98	0.59	0.66	0.48	0.78	0.65	0.79	0.59	0.66	0.57	0.53	0.62	0.73	0.56	0.58	0.59	0.53	0.38	0.60
	-3.1	SPRR1A	-0.79	-0.67	-0.59	-0.56	-0.75	-0.61	-0.66	0.68	0.58	0.58	0.60	0.56	0.63	0.65	0.58	0.60	0.76	0.57	0.64	0.65	0.77	0.71	0.58	0.74	0.44	0.56	0.79	0.68	0.93	0.64	0.76	0.76	0.73	0.75	0.74	0.49	0.73	0.90	0.78	0.67	0.77	0.74	0.55	0.80	0.72	0.81	0.75	0.78	0.68	0.66	0.62
	-3.3	KLHL31	-0.30	-0.56	-0.54	-0.49	-0.56	-0.50	-0.57	0.71	0.89	0.85	0.84	0.89	0.80	0.70	0.83	0.87	0.55	0.82	0.74	0.80	0.45	0.47	0.89	0.41	0.91	0.74	0.40	0.49	0.39	0.71	0.40	0.40	0.39	0.45	0.40	0.90	0.45	0.40	0.40	0.41	0.30	0.36	0.87	0.43	0.43	0.37	0.38	0.31	0.40	0.28	0.78
TG	-3.4	ALDOC	-0.70	-0.67	-0.59	-0.56	-0.66	-0.58	-0.66	0.64	0.57	0.54	0.56	0.52	0.58	0.57	0.50	0.54	0.59	0.51	0.62	0.59	0.59	0.55	0.54	0.62	0.47	0.59	0.86	0.51	0.81	0.54	0.58	0.62	0.57	0.90	0.58	0.44	0.63	0.76	0.67	0.65	0.66	0.65	0.57	0.68	0.74	0.93	0.75	0.68	0.70	0.72	0.64
TG	-3.4	ALDOB	-0.70	-0.62	-0.57	-0.54	-0.65	-0.65	-0.63	0.61	0.56	0.48	0.49	0.49	0.53	0.52	0.49	0.50	0.55	0.47	0.57	0.53	0.55	0.52	0.51	0.56	0.46	0.49	0.86	0.43	0.80	0.47	0.49	0.58	0.48	0.92	0.55	0.45	0.59	0.79	0.61	0.71	0.69	0.65	0.56	0.68	0.72	0.96	0.77	0.60	0.69	0.80	0.58
TG	-5.5	GLYATL1	-0.80	-0.67	-0.61	-0.59	-0.75	-0.71	-0.68	0.67	0.58	0.52	0.56	0.53	0.57	0.57	0.56	0.55	0.72	0.49	0.60	0.56	0.72	0.69	0.52	0.72	0.43	0.49	0.78	0.61	0.97	0.54	0.67	0.72	0.63	0.80	0.70	0.44	0.70	1.00	0.72	0.74	0.88	0.76	0.57	0.77	0.78	0.81	0.75	0.73	0.75	0.77	0.60
	-20.0	HIST1H1A	-0.57	-0.74	-0.63	-0.59	-0.78	-0.73	-0.61	0.69	0.92	0.83	0.81	0.90	0.77	0.68	0.95	0.90	0.63	0.65	0.73	0.76	0.60	0.62	0.82	0.61	0.84	0.63	0.66	0.52	0.61	0.69	0.50	0.60	0.56	0.64	0.54	0.85	0.61	0.59	0.55	0.60	0.48	0.64	0.89	0.54	0.59	0.58	0.50	0.49	0.48	0.33	0.73
TG	20.3	TOP2A	0.73	0.78	0.66	0.62	0.96	0.90	0.66	-0.71	-0.79	-0.70	-0.70	-0.75	-0.70	-0.66	-0.78	-0.75	-0.68	-0.61	-0.69	-0.68	-0.70	-0.70	-0.69	-0.72	-0.65	-0.60	-0.74	-0.60	-0.73	-0.64	-0.61	-0.70	-0.67	-0.72	-0.68	-0.66	-0.72	-0.66	-0.76	-0.68	-0.67	-0.76	-0.62	-0.69	-0.68	[illegible]	-0.64	-0.55	-0.62	-0.51	-0.71
TG	19.4	CDC25C	0.68	0.80	0.63	0.59	0.96	0.84	0.63	-0.69	-0.77	-0.70	-0.69	-0.74	-0.69	-0.66	-0.78	-0.75	-0.68	-0.62	-0.66	-0.68	-0.70	-0.70	-0.68	-0.70	-0.64	-0.57	-0.67	-0.64	-0.69	-0.62	-0.64	-0.70	-0.66	-0.65	-0.69	-0.66	-0.64	-0.66	-0.67	-0.66	-0.64	-0.63	-0.73	-0.61	-0.62	-0.61	-0.56	-0.54	-0.60	-0.43	-0.68
TG	18.9	CDKN3	0.76	0.80	0.69	0.65	0.97	0.87	0.70	-0.75	-0.78	-0.72	-0.71	-0.74	-0.73	-0.71	-0.77	-0.75	-0.70	-0.64	-0.71	-0.71	-0.72	-0.70	-0.70	-0.75	-0.62	-0.62	-0.77	-0.62	-0.78	-0.68	-0.66	-0.72	-0.70	-0.75	-0.70	-0.64	-0.72	-0.76	-0.70	-0.75	-0.70	-0.68	-0.76	-0.66	-0.72	-0.72	-0.65	-0.59	-0.63	-0.52	-0.73
TG	18.9	MELK	0.74	0.78	0.69	0.64	0.96	0.89	0.68	-0.70	-0.77	-0.69	-0.70	-0.74	-0.70	-0.67	-0.78	-0.74	-0.70	-0.61	-0.68	-0.68	-0.72	-0.71	-0.68	-0.73	-0.64	-0.58	-0.74	-0.62	-0.74	-0.63	-0.64	-0.72	-0.68	-0.72	-0.70	-0.65	-0.68	-0.73	-0.68	-0.73	-0.70	-0.67	-0.74	-0.65	-0.69	-0.68	-0.63	-0.55	-0.61	-0.51	-0.70
TG	18.5	NUF2	0.75	0.79	0.66	0.62	0.97	0.90	0.68	-0.72	-0.77	-0.69	-0.68	-0.73	-0.71	-0.68	-0.76	-0.73	-0.70	-0.62	-0.69	-0.68	-0.72	-0.71	-0.67	-0.75	-0.62	-0.59	-0.76	-0.62	-0.77	-0.64	-0.65	-0.72	-0.70	-0.73	-0.71	-0.64	-0.69	-0.74	-0.70	-0.76	-0.71	-0.67	-0.74	-0.65	-0.70	-0.70	-0.65	-0.55	-0.64	-0.55	-0.72
TG	17.4	CENPF	0.73	0.77	0.66	0.61	0.95	0.85	0.66	-0.75	-0.77	-0.71	-0.71	-0.74	-0.73	-0.70	-0.75	-0.74	-0.70	-0.65	-0.70	-0.70	-0.73	-0.71	-0.69	-0.77	-0.61	-0.62	-0.74	-0.64	-0.76	-0.66	-0.65	-0.72	-0.72	-0.72	-0.71	-0.63	-0.68	-0.74	-0.69	-0.75	-0.70	-0.64	-0.74	-0.64	-0.72	-0.68	-0.65	-0.54	-0.63	-0.52	-0.73
TG	16.4	KIF20A	0.71	0.80	0.66	0.62	0.96	0.86	0.64	-0.74	-0.78	-0.72	-0.70	-0.74	-0.73	-0.70	-0.76	-0.75	-0.63	-0.64	-0.70	-0.70	-0.67	-0.66	-0.70	-0.73	-0.64	-0.63	-0.74	-0.59	-0.72	-0.67	-0.60	-0.67	-0.69	-0.71	-0.65	-0.63	-0.69	-0.70	-0.66	-0.74	-0.66	-0.61	-0.77	-0.58	-0.70	-0.66	-0.60	-0.51	-0.61	-0.47	-0.73
TG	16.4	EXO1	0.76	0.82	0.70	0.67	0.97	0.89	0.70	-0.71	-0.77	-0.69	-0.69	-0.73	-0.70	-0.67	-0.76	-0.74	-0.70	-0.61	-0.69	-0.68	-0.72	-0.70	-0.67	-0.75	-0.63	-0.60	-0.76	-0.61	-0.75	-0.64	-0.63	-0.72	-0.71	-0.73	-0.71	-0.64	-0.69	-0.73	-0.69	-0.75	-0.70	-0.67	-0.75	-0.65	-0.70	-0.71	-0.64	-0.57	-0.64	-0.53	-0.71
TG	15.4	KIF4A	0.75	0.79	0.70	0.65	0.97	0.88	0.71	-0.71	-0.77	-0.68	-0.69	-0.73	-0.69	-0.66	-0.76	-0.72	-0.70	-0.63	-0.67	-0.67	-0.71	-0.71	-0.67	-0.74	-0.64	-0.59	-0.74	-0.63	-0.76	-0.62	-0.63	-0.72	-0.70	-0.73	-0.72	-0.66	-0.67	-0.74	-0.67	-0.73	-0.71	-0.69	-0.74	-0.67	-0.68	-0.70	-0.61	-0.58	-0.65	-0.55	-0.69
TG	15.0	BUB1	0.77	0.79	0.71	0.67	0.97	0.89	0.70	-0.73	-0.76	-0.68	-0.68	-0.72	-0.70	-0.68	-0.75	-0.72	-0.66	-0.61	-0.69	-0.67	-0.70	-0.69	-0.67	-0.76	-0.61	-0.60	-0.77	-0.60	-0.76	-0.64	-0.62	-0.69	-0.72	-0.75	-0.68	-0.62	-0.74	-0.75	-0.69	-0.76	-0.72	-0.66	-0.74	-0.64	-0.73	-0.71	-0.65	-0.56	-0.65	-0.54	-0.71
TG	14.5	TTK	0.74	0.81	0.66	0.62	0.96	0.88	0.67	-0.73	-0.76	-0.69	-0.69	-0.72	-0.71	-0.69	-0.76	-0.73	-0.72	-0.63	-0.69	-0.69	-0.73	-0.72	-0.68	-0.74	-0.61	-0.60	-0.75	-0.64	-0.76	-0.65	-0.66	-0.73	-0.69	-0.74	-0.71	-0.63	-0.69	-0.73	-0.71	-0.73	-0.69	-0.68	-0.73	-0.67	-0.70	-0.70	-0.67	-0.59	-0.61	-0.53	-0.73
TG	14.5	ERCC6L	0.77	0.79	0.68	0.64	0.96	0.87	0.71	-0.71	-0.77	-0.67	-0.67	-0.72	-0.68	-0.65	-0.75	-0.72	-0.68	-0.60	-0.67	-0.66	-0.70	-0.68	-0.66	-0.72	-0.63	-0.58	-0.76	-0.59	-0.76	-0.61	-0.62	-0.70	-0.67	-0.75	-0.69	-0.64	-0.67	-0.75	-0.67	-0.76	-0.72	-0.69	-0.75	-0.65	-0.70	-0.72	-0.65	-0.58	-0.66	-0.57	-0.70
TG	14.5	ANLN	0.77	0.78	0.70	0.66	0.95	0.88	0.70	-0.72	-0.77	-0.70	-0.68	-0.73	-0.70	-0.68	-0.77	-0.73	-0.67	-0.60	-0.68	-0.68	-0.71	-0.71	-0.68	-0.77	-0.62	-0.58	-0.74	-0.61	-0.75	-0.63	-0.62	-0.71	-0.71	-0.74	-0.68	-0.63	-0.70	-0.74	-0.69	-0.76	-0.70	-0.66	-0.75	-0.63	-0.72	-0.69	-0.63	-0.54	-0.64	-0.53	-0.70
TG	14.5	CCNB2	0.76	0.81	0.69	0.65	0.98	0.88	0.69	-0.73	-0.75	-0.69	-0.69	-0.72	-0.70	-0.68	-0.75	-0.72	-0.69	-0.62	-0.69	-0.68	-0.72	-0.71	-0.67	-0.77	-0.61	-0.61	-0.75	-0.64	-0.76	-0.65	-0.66	-0.72	-0.73	-0.74	-0.71	-0.62	-0.73	-0.74	-0.71	-0.74	-0.72	-0.65	-0.73	-0.65	-0.71	-0.70	-0.64	-0.58	-0.64	-0.53	-0.72
TG	14.4	NEK2	0.77	0.78	0.65	0.60	0.96	0.86	0.67	-0.71	-0.75	-0.67	-0.67	-0.72	-0.69	-0.66	-0.74	-0.72	-0.73	-0.61	-0.67	-0.67	-0.75	-0.74	-0.66	-0.75	-0.60	-0.57	-0.77	-0.66	-0.79	-0.61	-0.67	-0.75	-0.71	-0.75	-0.75	-0.64	-0.65	-0.77	-0.70	-0.77	-0.74	-0.71	-0.72	-0.69	-0.71	-0.73	-0.66	-0.60	-0.68	-0.59	-0.68
TG	14.4	E2F7	0.73	0.76	0.63	0.59	0.95	0.89	0.65	-0.68	-0.76	-0.68	-0.68	-0.72	-0.69	-0.67	-0.76	-0.74	-0.71	-0.60	-0.68	-0.67	-0.70	-0.69	-0.67	-0.70	-0.61	-0.57	-0.73	-0.60	-0.75	-0.64	-0.64	-0.71	-0.66	-0.70	-0.69	-0.65	-0.67	-0.73	-0.65	-0.70	-0.68	-0.68	-0.73	-0.65	-0.63	-0.67	-0.61	-0.58	-0.60	-0.50	-0.68
TG	13.9	NCAPG	0.75	0.82	0.73	0.69	0.97	0.86	0.71	-0.72	-0.78	-0.71	-0.71	-0.75	-0.72	-0.69	-0.78	-0.76	-0.68	-0.64	-0.70	-0.70	-0.70	-0.67	-0.70	-0.74	-0.67	-0.61	-0.77	-0.61	-0.76	-0.66	-0.63	-0.70	-0.71	-0.75	-0.69	-0.67	-0.72	-0.73	-0.68	-0.73	-0.69	-0.65	-0.76	-0.65	-0.69	-0.72	-0.61	-0.57	-0.65	-0.51	-0.71
TG	13.3	RRM2	0.69	0.77	0.71	0.67	0.94	0.87	0.69	-0.72	-0.77	-0.70	-0.68	-0.72	-0.70	-0.67	-0.75	-0.72	-0.62	-0.61	-0.69	-0.68	-0.64	-0.63	-0.68	-0.71	-0.64	-0.60	-0.72	-0.55	-0.70	-0.64	-0.56	-0.65	-0.68	-0.70	-0.64	-0.65	-0.68	-0.68	-0.65	-0.70	-0.66	-0.61	-0.77	-0.57	-0.67	-0.65	-0.55	-0.50	-0.59	-0.47	-0.70
	13.3	DEPDC1	0.72	0.76	0.61	0.55	0.94	0.88	0.63	-0.67	-0.74	-0.64	-0.64	-0.71	-0.65	-0.61	-0.75	-0.71	-0.68	-0.57	-0.62	-0.63	-0.69	-0.69	-0.64	-0.69	-0.62	-0.53	-0.69	-0.60	-0.70	-0.56	-0.59	-0.69	-0.65	-0.68	-0.68	-0.64	-0.62	-0.69	-0.65	-0.73	-0.67	-0.67	-0.71	-0.60	-0.63	-0.64	-0.60	-0.55	-0.62	-0.51	-0.65
TG	13.2	BUB1B	0.74	0.75	0.69	0.64	0.94	0.84	0.65	-0.74	-0.77	-0.73	-0.72	-0.76	-0.74	-0.71	-0.78	-0.76	-0.68	-0.65	-0.71	-0.71	-0.73	-0.72	-0.71	-0.78	-0.63	-0.61	-0.72	-0.66	-0.72	-0.67	-0.65	-0.71	-0.74	-0.70	-0.69	-0.65	-0.72	-0.70	-0.70	-0.74	-0.68	-0.64	-0.74	-0.63	-0.72	-0.65	-0.64	-0.53	-0.59	-0.46	-0.72
TG	12.8	SPC25	0.74	0.80	0.69	0.66	0.96	0.87	0.71	-0.71	-0.77	-0.69	-0.69	-0.73	-0.70	-0.67	-0.76	-0.74	-0.69	-0.62	-0.68	-0.68	-0.70	-0.68	-0.68	-0.71	-0.65	-0.59	-0.74	-0.60	-0.74	-0.64	-0.62	-0.70	-0.67	-0.72	-0.69	-0.66	-0.66	-0.73	-0.67	-0.73	-0.69	-0.67	-0.76	-0.66	-0.67	-0.70	-0.60	-0.58	-0.65	-0.53	-0.70
TG	12.8	PBK	0.71	0.76	0.67	0.62	0.96	0.88	0.68	-0.69	-0.78	-0.69	-0.69	-0.75	-0.69	-0.65	-0.78	-0.74	-0.67	-0.61	-0.67	-0.66	-0.68	-0.68	-0.68	-0.70	-0.66	-0.58	-0.72	-0.59	-0.72	-0.61	-0.59	-0.68	-0.65	-0.71	-0.68	-0.68	-0.64	-0.71	-0.64	-0.72	-0.66	-0.64	-0.76	-0.62	-0.66	-0.67	-0.59	-0.51	-0.63	-0.52	-0.68
TG	11.7	ASPM	0.71	0.75	0.59	0.54	0.94	0.88	0.61	-0.70	-0.74	-0.68	-0.67	-0.71	-0.69	-0.67	-0.73	-0.71	-0.71	-0.62	-0.67	-0.67	-0.71	-0.71	-0.66	-0.70	-0.59	-0.58	-0.72	-0.63	-0.72	-0.63	-0.63	-0.72	-0.65	-0.69	-0.72	-0.62	-0.63	-0.71	-0.66	-0.73	-0.66	-0.66	-0.71	-0.64	-0.66	-0.66	-0.64	-0.54	-0.61	-0.53	-0.70
TG	11.7	KIF18A	0.76	0.78	0.66	0.61	0.96	0.90	0.68	-0.72	-0.76	-0.68	-0.67	-0.73	-0.69	-0.67	-0.75	-0.72	-0.67	-0.61	-0.68	-0.67	-0.69	-0.68	-0.67	-0.71	-0.62	-0.60	-0.73	-0.60	-0.74	-0.62	-0.63	-0.69	-0.65	-0.73	-0.67	-0.61	-0.72	-0.73	-0.69	-0.77	-0.71	-0.68	-0.74	-0.64	-0.70	-0.69	-0.65	-0.57	-0.63	-0.53	-0.72
TG	10.7	GPSM2	0.77	0.77	0.70	0.65	0.96	0.88	0.71	-0.71	-0.78	-0.69	-0.70	-0.75	-0.69	-0.66	-0.78	-0.74	-0.71	-0.62	-0.67	-0.68	-0.73	-0.73	-0.69	-0.75	-0.65	-0.57	-0.72	-0.65	-0.75	-0.61	-0.64	-0.74	-0.71	-0.72	-0.72	-0.67	-0.68	-0.74	-0.68	-0.73	-0.71	-0.70	-0.75	-0.66	-0.69	-0.68	-0.62	-0.57	-0.64	-0.53	-0.68
TG	10.4	CDC20	0.80	0.80	0.71	0.66	0.95	0.85	0.69	-0.75	-0.75	-0.71	-0.70	-0.73	-0.72	-0.70	-0.76	-0.74	-0.73	-0.63	-0.70	-0.71	-0.77	-0.75	-0.70	-0.80	-0.60	-0.59	-0.76	-0.69	-0.79	-0.66	-0.70	-0.75	-0.76	-0.74	-0.74	-0.62	-0.75	-0.77	-0.74	-0.75	-0.75	-0.69	-0.72	-0.68	-0.77	-0.70	-0.68	-0.60	-0.64	-0.53	-0.72
TG	10.3	ARHGEF39	0.73	0.78	0.61	0.55	0.94	0.84	0.61	-0.72	-0.74	-0.69	-0.70	-0.71	-0.71	-0.70	-0.73	-0.72	-0.75	-0.64	-0.68	-0.69	-0.77	-0.74	-0.67	-0.72	-0.59	-0.61	-0.76	-0.70	-0.76	-0.65	-0.71	-0.77	-0.68	-0.73	-0.75	-0.62	-0.65	-0.74	-0.71	-0.73	-0.71	-0.69	-0.70	-0.69	-0.73	-0.72	-0.69	-0.62	-0.63	-0.54	-0.71
TG	10.3	CCNA2	0.73	0.76	0.66	0.61	0.96	0.89	0.65	-0.70	-0.74	-0.67	-0.68	-0.71	-0.68	-0.67	-0.74	-0.71	-0.68	-0.60	-0.66	-0.67	-0.71	-0.68	-0.67	-0.74	-0.60	-0.57	-0.74	-0.63	-0.76	-0.62	-0.65	-0.72	-0.69	-0.73	-0.69	-0.61	-0.70	-0.75	-0.70	-0.71	-0.72	-0.66	-0.71	-0.63	-0.70	-0.70	-0.64	-0.57	-0.62	-0.53	-0.69
TG	10.1	MKI67	0.74	0.79	0.73	0.69	0.96	0.83	0.70	-0.71	-0.73	-0.66	-0.69	-0.69	-0.68	-0.67	-0.72	-0.70	-0.65	-0.62	-0.66	-0.67	-0.69	-0.66	-0.66	-0.77	-0.59	-0.57	-0.74	-0.59	-0.78	-0.64	-0.61	-0.69	-0.73	-0.73	-0.67	-0.62	-0.71	-0.75	-0.66	-0.68	-0.71	-0.64	-0.70	-0.65	-0.68	-0.69	-0.58	-0.56	-0.68	-0.54	-0.66
TG	9.9	HMMR	0.69	0.81	0.62	0.58	0.93	0.86	0.62	-0.67	-0.77	-0.70	-0.67	-0.73	-0.69	-0.64	-0.77	-0.74	-0.63	-0.58	-0.66	-0.66	-0.66	-0.66	-0.68	-0.67	-0.65	-0.58	-0.68	-0.59	-0.64	-0.61	-0.59	-0.66	-0.64	-0.65	-0.64	-0.64	-0.64	-0.62	-0.64	-0.69	-0.59	-0.62	-0.75	-0.56	-0.63	-0.61	-0.56	-0.52	-0.56	-0.40	-0.69
TG	9.7	POLQ	0.75	0.79	0.67	0.63	0.96	0.89	0.67	-0.67	-0.72	-0.65	-0.67	-0.69	-0.66	-0.64	-0.74	-0.70	-0.72	-0.58	-0.65	-0.65	-0.75	-0.72	-0.64	-0.74	-0.58	-0.54	-0.76	-0.64	-0.77	-0.61	-0.68	-0.76	-0.71	-0.73	-0.74	-0.62	-0.71	-0.75	-0.73	-0.69	-0.71	-0.71	-0.69	-0.69	-0.69	-0.72	-0.64	-0.63	-0.63	-0.53	-0.66
TG	9.5	CDC6	0.74	0.80	0.70	0.67	0.93	0.86	0.71	-0.68	-0.74	-0.64	-0.67	-0.70	-0.65	-0.63	-0.73	-0.69	-0.69	-0.59	-0.66	-0.64	-0.69	-0.66	-0.64	-0.75	-0.60	-0.57	-0.72	-0.58	-0.75	-0.61	-0.61	-0.68	-0.70	-0.71	-0.67	-0.63	-0.67	-0.74	-0.68	-0.71	-0.70	-0.68	-0.71	-0.68	-0.66	-0.69	-0.62	-0.61	-0.63	-0.53	-0.66
TG	9.3	DTL	0.72	0.76	0.62	0.56	0.91	0.83	0.61	-0.72	-0.73	-0.69	-0.69	-0.72	-0.71	-0.69	-0.73	-0.73	-0.75	-0.65	-0.70	-0.69	-0.75	-0.76	-0.68	-0.73	-0.59	-0.60	-0.66	-0.69	-0.70	-0.65	-0.68	-0.74	-0.69	-0.64	-0.73	-0.63	-0.60	-0.69	-0.67	-0.71	-0.67	-0.66	-0.69	-0.65	-0.68	-0.61	-0.65	-0.55	-0.59	-0.47	-0.71
TG	9.3	KIF23	0.75	0.73	0.72	0.68	0.94	0.84	0.69	-0.74	-0.76	-0.72	-0.72	-0.75	-0.72	-0.71	-0.77	-0.74	-0.67	-0.63	-0.70	-0.70	-0.74	-0.73	-0.70	-0.79	-0.61	-0.59	-0.71	-0.65	-0.73	-0.66	-0.64	-0.72	-0.76	-0.68	-0.70	-0.63	-0.71	-0.72	-0.69	-0.72	-0.70	-0.66	-0.73	-0.64	-0.71	-0.64	-0.61	-0.56	-0.62	-0.50	-0.69
TG	9.2	CENPM	0.81	0.82	0.72	0.69	0.95	0.87	0.70	-0.71	-0.72	-0.67	-0.68	-0.69	-0.69	-0.68	-0.73	-0.70	-0.72	-0.59	-0.69	-0.68	-0.76	-0.73	-0.66	-0.78	-0.58	-0.57	-0.76	-0.66	-0.79	-0.64	-0.70	-0.76	-0.74	-0.75	-0.74	-0.59	-0.76	-0.77	-0.75	-0.72	-0.75	-0.72	-0.70	-0.72	-0.74	-0.73	-0.67	-0.66	-0.67	-0.55	-0.69
TG	8.7	CEP55	0.79	0.77	0.77	0.73	0.95	0.86	0.75	-0.73	-0.74	-0.66	-0.67	-0.70	-0.67	-0.65	-0.73	-0.68	-0.65	-0.60	-0.66	-0.65	-0.69	-0.69	-0.66	-0.79	-0.61	-0.58	-0.75	-0.59	-0.79	-0.61	-0.59	-0.69	-0.73	-0.76	-0.67	-0.61	-0.74	-0.78	-0.69	-0.77	-0.75	-0.66	-0.73	-0.64	-0.75	-0.70	-0.63	-0.55	-0.69	-0.59	-0.69
TG	8.5	PRC1	0.70	0.72	0.68	0.63	0.94	0.85	0.65	-0.71	-0.74	-0.70	-0.68	-0.72	-0.70	-0.68	-0.75	-0.72	-0.65	-0.62	-0.66	-0.68	-0.70	-0.70	-0.68	-0.75	-0.61	-0.57	-0.66	-0.63	-0.68	-0.63	-0.63	-0.69	-0.71	-0.65	-0.67	-0.62	-0.69	-0.67	-0.67	-0.70	-0.65	-0.61	-0.72	-0.59	-0.66	-0.58	-0.58	-0.49	-0.57	-0.44	-0.69
TG	8.4	HJURP	0.80	0.74	0.66	0.63	0.94	0.86	0.68	-0.64	-0.65	-0.56	-0.56	-0.60	-0.58	-0.56	-0.64	-0.61	-0.63	-0.49	-0.55	-0.56	-0.69	-0.66	-0.55	-0.73	-0.51	-0.45	-0.76	-0.56	-0.77	-0.51	-0.60	-0.69	-0.67	-0.74	-0.69	-0.51	-0.69	-0.76	-0.66	-0.75	-0.71	-0.66	-0.64	-0.63	-0.70	-0.72	-0.64	-0.54	-0.67	-0.60	-0.59
TG	8.2	CDC45	0.77	0.81	0.73	0.69	0.95	0.88	0.72	-0.70	-0.73	-0.65	-0.68	-0.69	-0.67	-0.65	-0.73	-0.69	-0.71	-0.60	-0.66	-0.66	-0.73	-0.71	-0.66	-0.75	-0.61	-0.56	-0.72	-0.64	-0.75	-0.61	-0.66	-0.73	-0.70	-0.72	-0.72	-0.62	-0.69	-0.73	-0.71	-0.69	-0.74	-0.70	-0.71	-0.70	-0.69	-0.71	-0.63	-0.64	-0.65	-0.55	-0.67
TG	7.9	CDCA3	0.76	0.80	0.64	0.60	0.96	0.90	0.69	-0.67	-0.71	-0.63	-0.63	-0.67	-0.64	-0.62	-0.71	-0.67	-0.74	-0.58	-0.63	-0.63	-0.74	-0.73	-0.62	-0.71	-0.57	-0.53	-0.76	-0.64	-0.79	-0.58	-0.67	-0.75	-0.65	-0.75	-0.77	-0.61	-0.66	-0.79	-0.69	-0.72	-0.76	-0.70	-0.70	-0.71	-0.69	-0.74	-0.65	-0.63	-0.69	-0.62	-0.67
TG	7.4	DLGAP5	0.78	0.78	0.67	0.64	0.95	0.88	0.67	-0.72	-0.72	-0.65	-0.64	-0.68	-0.68	-0.66	-0.72	-0.70	-0.65	-0.58	-0.66	-0.65	-0.69	-0.66	-0.64	-0.75	-0.55	-0.55	-0.75	-0.57	-0.79	-0.62	-0.61	-0.68	-0.69	-0.74	-0.67	-0.58	-0.71	-0.77	-0.66	-0.76	-0.75	-0.64	-0.70	-0.62	-0.72	-0.71	-0.63	-0.54	-0.65	-0.57	-0.68
TG	7.4	CHEK1	0.75	0.76	0.68	0.65	0.93	0.88	0.67	-0.69	-0.74	-0.65	-0.65	-0.70	-0.66	-0.63	-0.74	-0.70	-0.63	-0.57	-0.65	-0.64	-0.67	-0.67	-0.64	-0.71	-0.60	-0.56	-0.73	-0.57	-0.72	-0.58	-0.57	-0.67	-0.65	-0.74	-0.66	-0.60	-0.68	-0.70	-0.63	-0.72	-0.69	-0.69	-0.71	-0.62	-0.69	-0.68	-0.63	-0.57	-0.60	-0.52	-0.66
	7.3	CENPK	0.67	0.76	0.64	0.61	0.90	0.87	0.67	-0.68	-0.74	-0.67	-0.64	-0.71	-0.66	-0.63	-0.74	-0.70	-0.61	-0.58	-0.63	-0.64	-0.63	-0.63	-0.65	-0.67	-0.61	-0.56	-0.65	-0.56	-0.66	-0.58	-0.61	-0.64	-0.63	-0.67	-0.61	-0.61	-0.67	-0.65	-0.66	-0.64	-0.62	-0.65	-0.71	-0.57	-0.63	-0.63	-0.59	-0.56	-0.60	-0.46	-0.68
TG	7.2	TRIP13	0.78	0.79	0.74	0.69	0.93	0.82	0.70	-0.74	-0.78	-0.71	-0.72	-0.76	-0.71	-0.68	-0.79	-0.76	-0.72	-0.63	-0.70	-0.70	-0.76	-0.75	-0.71	-0.78	-0.65	-0.59	-0.73	-0.68	-0.75	-0.64	-0.67	-0.74	-0.73	-0.72	-0.72	-0.67	-0.70	-0.75	-0.71	-0.75	-0.73	-0.69	-0.75	-0.68	-0.75	-0.68	-0.66	-0.58	-0.65	-0.54	-0.70
TG	7.1	UBE2C	0.78	0.78	0.63	0.58	0.93	0.85	0.63	-0.76	-0.73	-0.68	-0.70	-0.71	-0.71	-0.70	-0.72	-0.73	-0.71	-0.63	-0.70	-0.70	-0.73	-0.71	-0.67	-0.77	-0.57	-0.60	-0.74	-0.67	-0.79	-0.66	-0.67	-0.71	-0.72	-0.73	-0.69	-0.60	-0.71	-0.79	-0.71	-0.77	-0.76	-0.64	-0.70	-0.66	-0.74	-0.71	-0.70	-0.57	-0.65	-0.58	-0.72
TG	6.9	NDC80	0.71	0.81	0.60	0.58	0.95	0.89	0.62	-0.64	-0.71	-0.62	-0.63	-0.67	-0.64	-0.61	-0.72	-0.68	-0.69	-0.55	-0.62	-0.61	-0.70	-0.67	-0.62	-0.69	-0.57	-0.53	-0.71	-0.57	-0.74	-0.59	-0.62	-0.70	-0.63	-0.68	-0.68	-0.59	-0.66	-0.72	-0.67	-0.71	-0.68	-0.65	-0.69	-0.62	-0.65	-0.68	-0.61	-0.57	-0.63	-0.51	-0.64
TG	6.9	KIF14	0.75	0.72	0.62	0.56	0.91	0.82	0.64	-0.68	-0.66	-0.61	-0.63	-0.65	-0.65	-0.65	-0.69	-0.66	-0.74	-0.59	-0.63	-0.64	-0.76	-0.74	-0.61	-0.76	-0.51	-0.53	-0.69	-0.67	-0.78	-0.60	-0.69	-0.75	-0.71	-0.69	-0.74	-0.56	-0.65	-0.76	-0.71	-0.72	-0.72	-0.68	-0.62	-0.70	-0.66	-0.67	-0.66	-0.59	-0.64	-0.57	-0.65
TG	6.5	PTTG1	0.74	0.79	0.67	0.63	0.97	0.87	0.67	-0.72	-0.75	-0.69	-0.68	-0.72	-0.70	-0.68	-0.74	-0.73	-0.65	-0.62	-0.67	-0.68	-0.69	-0.69	-0.68	-0.73	-0.62	-0.58	-0.73	-0.63	-0.74	-0.62	-0.62	-0.70	-0.69	-0.73	-0.69	-0.63	-0.69	-0.73	-0.67	-0.73	-0.70	-0.65	-0.74	-0.62	-0.70	-0.68	-0.60	-0.53	-0.67	-0.53	-0.70
TG	6.5	KIF2C	0.77	0.79	0.72	0.68	0.95	0.87	0.70	-0.73	-0.75	-0.69	-0.69	-0.72	-0.70	-0.67	-0.75	-0.72	-0.67	-0.61	-0.69	-0.68	-0.71	-0.70	-0.68	-0.78	-0.62	-0.57	-0.72	-0.63	-0.74	-0.62	-0.63	-0.69	-0.73	-0.72	-0.69	-0.64	-0.72	-0.74	-0.69	-0.76	-0.74	-0.65	-0.74	-0.63	-0.74	-0.66	-0.63	-0.53	-0.65	-0.53	-0.71
TG	6.4	KIFC1	0.77	0.74	0.66	0.61	0.94	0.81	0.65	-0.72	-0.72	-0.68	-0.68	-0.71	-0.70	-0.69	-0.72	-0.71	-0.70	-0.63	-0.66	-0.68	-0.73	-0.71	-0.66	-0.76	-0.57	-0.57	-0.72	-0.67	-0.76	-0.63	-0.68	-0.72	-0.71	-0.71	-0.72	-0.60	-0.69	-0.75	-0.68	-0.71	-0.72	-0.66	-0.69	-0.67	-0.72	-0.67	-0.63	-0.56	-0.64	-0.51	-0.68
TG	6.3	SHCBP1	0.79	0.79	0.76	0.74	0.92	0.87	0.77	-0.64	-0.67	-0.56	-0.58	-0.62	-0.58	-0.54	-0.68	-0.61	-0.61	-0.48	-0.59	-0.56	-0.65	-0.62	-0.57	-0.74	-0.54	-0.48	-0.77	-0.49	-0.78	-0.53	-0.57	-0.66	-0.68	-0.77	-0.64	-0.56	-0.72	-0.76	-0.68	-0.74	-0.74	-0.65	-0.67	-0.61	-0.71	-0.73	-0.58	-0.55	-0.69	-0.61	-0.61
TG	6.2	ZWINT	0.67	0.74	0.70	0.67	0.94	0.85	0.67	-0.70	-0.74	-0.67	-0.66	-0.70	-0.68	-0.66	-0.72	-0.70	-0.59	-0.61	-0.65	-0.66	-0.63	-0.62	-0.66	-0.71	-0.61	-0.57	-0.70	-0.55	-0.67	-0.62	-0.56	-0.62	-0.67	-0.69	-0.63	-0.62	-0.66	-0.66	-0.61	-0.67	-0.61	-0.56	-0.73	-0.57	-0.64	-0.63	-0.52	-0.47	-0.60	-0.46	-0.68
TG	6.0	ORC1	0.78	0.77	0.70	0.67	0.91	0.86	0.66	-0.69	-0.69	-0.63	-0.65	-0.66	-0.65	-0.63	-0.71	-0.68	-0.67	-0.54	-0.65	-0.63	-0.73	-0.72	-0.63	-0.78	-0.55	-0.53	-0.70	-0.63	-0.73	-0.59	-0.64	-0.73	-0.73	-0.69	-0.71	-0.56	-0.70	-0.73	-0.70	-0.72	-0.72	-0.70	-0.67	-0.64	-0.71	-0.65	-0.64	-0.61	-0.60	-0.50	-0.66
TG	6.0	FAM72C	0.78	0.76	0.63	0.59	0.94	0.88	0.67	-0.68	-0.70	-0.63	-0.62	-0.67	-0.65	-0.63	-0.72	-0.67	-0.72	-0.56	-0.64	-0.63	-0.75	-0.73	-0.62	-0.76	-0.55	-0.52	-0.75	-0.63	-0.80	-0.59	-0.66	-0.75	-0.70	-0.73	-0.74	-0.59	-0.70	-0.78	-0.72	-0.76	-0.75	-0.71	-0.68	-0.70	-0.70	-0.71	-0.67	-0.59	-0.69	-0.61	-0.66
	5.9	MND1	0.69	0.75	0.62	0.59	0.92	0.89	0.64	-0.68	-0.76	-0.66	-0.65	-0.72	-0.66	-0.62	-0.77	-0.71	-0.59	-0.56	-0.61	-0.63	-0.62	-0.63	-0.64	-0.68	-0.61	-0.53	-0.66	-0.54	-0.67	-0.55	-0.54	-0.62	-0.63	-0.67	-0.61	-0.62	-0.63	-0.66	-0.62	-0.69	-0.65	-0.63	-0.74	-0.52	-0.65	-0.61	-0.58	-0.49	-0.60	-0.49	-0.67
TG	5.9	FAM72D	0.78	0.76	0.64	0.60	0.94	0.87	0.66	-0.67	-0.69	-0.61	-0.62	-0.66	-0.64	-0.63	-0.71	-0.67	-0.70	-0.55	-0.63	-0.62	-0.74	-0.71	-0.61	-0.77	-0.54	-0.51	-0.75	-0.61	-0.80	-0.58	-0.65	-0.74	-0.71	-0.74	-0.73	-0.58	-0.70	-0.78	-0.71	-0.76	-0.75	-0.71	-0.66	-0.69	-0.70	-0.72	-0.66	-0.59	-0.70	-0.61	-0.64
TG	5.8	MTFR2	0.80	0.80	0.64	0.60	0.90	0.86	0.68	-0.67	-0.71	-0.61	-0.64	-0.67	-0.63	-0.61	-0.73	-0.68	-0.75	-0.54	-0.65	-0.62	-0.75	-0.73	-0.61	-0.75	-0.55	-0.53	-0.77	-0.63	-0.80	-0.58	-0.66	-0.75	-0.68	-0.76	-0.72	-0.61	-0.66	-0.79	-0.72	-0.76	-0.75	-0.76	-0.67	-0.72	-0.70	-0.75	-0.70	-0.66	-0.66	-0.62	-0.65
TG	5.6	ESCO2	0.70	0.74	0.64	0.60	0.91	0.85	0.67	-0.63	-0.68	-0.59	-0.61	-0.63	-0.60	-0.58	-0.67	-0.63	-0.66	-0.55	-0.61	-0.59	-0.70	-0.66	-0.59	-0.66	-0.56	-0.53	-0.69	-0.58	-0.71	-0.56	-0.59	-0.69	-0.62	-0.69	-0.69	-0.56	-0.59	-0.71	-0.64	-0.70	-0.68	-0.61	-0.65	-0.65	-0.64	-0.68	-0.61	-0.55	-0.61	-0.56	-0.61
TG	5.6	C5orf34	0.69	0.75	0.59	0.54	0.91	0.88	0.62	-0.68	-0.75	-0.66	-0.66	-0.71	-0.67	-0.63	-0.76	-0.71	-0.73	-0.57	-0.66	-0.64	-0.74	-0.74	-0.64	-0.69	-0.60	-0.55	-0.76	-0.63	-0.74	-0.60	-0.64	-0.75	-0.63	-0.72	-0.74	-0.63	-0.65	-0.73	-0.70	-0.70	-0.69	-0.71	-0.73	-0.67	-0.66	-0.68	-0.62	-0.61	-0.61	-0.52	-0.69
TG	5.5	UBE2T	0.77	0.80	0.68	0.64	0.95	0.89	0.69	-0.72	-0.74	-0.67	-0.67	-0.70	-0.69	-0.67	-0.73	-0.71	-0.72	-0.61	-0.67	-0.67	-0.73	-0.72	-0.66	-0.77	-0.60	-0.58	-0.74	-0.64	-0.79	-0.62	-0.66	-0.73	-0.72	-0.74	-0.72	-0.62	-0.69	-0.77	-0.70	-0.77	-0.73	-0.69	-0.72	-0.68	-0.70	-0.70	-0.66	-0.58	-0.64	-0.57	-0.70
TG	5.4	ORC6	0.77	0.81	0.75	0.71	0.92	0.88	0.72	-0.71	-0.73	-0.65	-0.67	-0.69	-0.67	-0.64	-0.73	-0.69	-0.69	-0.60	-0.68	-0.65	-0.71	-0.70	-0.66	-0.77	-0.61	-0.57	-0.76	-0.61	-0.76	-0.62	-0.63	-0.71	-0.73	-0.76	-0.70	-0.62	-0.72	-0.75	-0.71	-0.73	-0.73	-0.68	-0.71	-0.67	-0.74	-0.73	-0.63	-0.59	-0.65	-0.57	-0.68
TG	5.4	HELLS	0.77	0.73	0.73	0.70	0.92	0.89	0.74	-0.69	-0.71	-0.63	-0.65	-0.67	-0.65	-0.62	-0.71	-0.67	-0.65	-0.57	-0.64	-0.63	-0.68	-0.67	-0.62	-0.73	-0.57	-0.53	-0.74	-0.56	-0.76	-0.58	-0.59	-0.69	-0.67	-0.74	-0.68	-0.60	-0.69	-0.75	-0.64	-0.70	-0.71	-0.71	-0.69	-0.65	-0.68	-0.71	-0.61	-0.58	-0.65	-0.59	-0.65
TG	5.4	FAM72A	0.76	0.75	0.64	0.60	0.94	0.87	0.65	-0.71	-0.73	-0.66	-0.66	-0.71	-0.68	-0.66	-0.74	-0.71	-0.70	-0.59	-0.66	-0.66	-0.74	-0.73	-0.65	-0.78	-0.58	-0.55	-0.74	-0.63	-0.77	-0.61	-0.63	-0.73	-0.72	-0.72	-0.71	-0.61	-0.69	-0.76	-0.69	-0.78	-0.74	-0.68	-0.70	-0.67	-0.72	-0.68	-0.66	-0.55	-0.68	-0.59	-0.68
TG	5.3	CENPL	0.72	0.74	0.64	0.58	0.92	0.85	0.67	-0.73	-0.78	-0.69	-0.70	-0.75	-0.71	-0.68	-0.77	-0.74	-0.75	-0.65	-0.69	-0.68	-0.75	-0.76	-0.69	-0.74	-0.63	-0.62	-0.70	-0.68	-0.73	-0.64	-0.64	-0.74	-0.70	-0.69	-0.74	-0.66	-0.63	-0.74	-0.67	-0.76	-0.68	-0.66	-0.74	-0.67	-0.69	-0.64	-0.66	-0.55	-0.63	-0.54	-0.72
TG	5.0	KIAA0101	0.77	0.82	0.73	0.70	0.94	0.87	0.75	-0.71	-0.72	-0.64	-0.66	-0.68	-0.66	-0.63	-0.71	-0.68	-0.71	-0.60	-0.65	-0.64	-0.72	-0.70	-0.64	-0.76	-0.60	-0.55	-0.73	-0.61	-0.78	-0.60	-0.62	-0.71	-0.72	-0.72	-0.72	-0.63	-0.67	-0.78	-0.70	-0.73	-0.74	-0.67	-0.71	-0.69	-0.69	-0.71	-0.59	-0.60	-0.70	-0.60	-0.67
TG	5.0	NUSAP1	0.70	0.74	0.65	0.60	0.94	0.87	0.63	-0.69	-0.73	-0.66	-0.67	-0.71	-0.67	-0.65	-0.73	-0.70	-0.66	-0.60	-0.65	-0.65	-0.69	-0.67	-0.65	-0.74	-0.58	-0.53	-0.67	-0.59	-0.72	-0.61	-0.61	-0.68	-0.69	-0.64	-0.67	-0.63	-0.64	-0.70	-0.64	-0.69	-0.69	-0.64	-0.69	-0.62	-0.65	-0.62	-0.57	-0.50	-0.62	-0.51	-0.66
TG	4.7	FANCB	0.76	0.76	0.67	0.64	0.91	0.89	0.73	-0.69	-0.70	-0.61	-0.64	-0.66	-0.63	-0.62	-0.71	-0.65	-0.70	-0.56	-0.65	-0.62	-0.72	-0.69	-0.60	-0.71	-0.54	-0.54	-0.74	-0.58	-0.80	-0.60	-0.64	-0.74	-0.66	-0.73	-0.72	-0.58	-0.66	-0.79	-0.71	-0.69	-0.76	-0.76	-0.68	-0.70	-0.71	-0.74	-0.66	-0.67	-0.66	-0.63	-0.67
TG	4.7	CENPW	0.77	0.81	0.69	0.65	0.92	0.86	0.73	-0.68	-0.72	-0.63	-0.68	-0.68	-0.65	-0.63	-0.74	-0.69	-0.76	-0.58	-0.66	-0.64	-0.77	-0.74	-0.64	-0.74	-0.58	-0.53	-0.75	-0.65	-0.80	-0.59	-0.68	-0.77	-0.68	-0.75	-0.76	-0.63	-0.66	-0.79	-0.73	-0.70	-0.76	-0.77	-0.69	-0.76	-0.70	-0.74	-0.66	-0.70	-0.66	-0.62	-0.66
TG	4.6	CDC25A	0.75	0.73	0.62	0.60	0.92	0.85	0.64	-0.66	-0.63	-0.57	-0.58	-0.58	-0.61	-0.61	-0.62	-0.60	-0.64	-0.55	-0.61	-0.59	-0.69	-0.66	-0.55	-0.71	-0.48	-0.52	-0.74	-0.58	-0.76	-0.58	-0.61	-0.69	-0.67	-0.73	-0.70	-0.52	-0.65	-0.74	-0.66	-0.70	-0.72	-0.64	-0.62	-0.66	-0.66	-0.73	-0.60	-0.59	-0.64	-0.60	-0.62
TG	4.5	RAD51	0.74	0.77	0.73	0.69	0.92	0.88	0.72	-0.71	-0.75	-0.67	-0.67	-0.72	-0.66	-0.62	-0.75	-0.70	-0.65	-0.59	-0.65	-0.64	-0.69	-0.69	-0.67	-0.75	-0.64	-0.54	-0.70	-0.61	-0.71	-0.57	-0.58	-0.67	-0.68	-0.72	-0.66	-0.64	-0.68	-0.72	-0.65	-0.74	-0.75	-0.67	-0.74	-0.64	-0.72	-0.66	-0.59	-0.55	-0.65	-0.55	-0.68
TG	4.2	FANCI	0.75	0.76	0.69	0.65	0.94	0.83	0.67	-0.67	-0.68	-0.63	-0.67	-0.67	-0.66	-0.66	-0.71	-0.68	-0.74	-0.60	-0.65	-0.66	-0.79	-0.74	-0.63	-0.78	-0.52	-0.52	-0.70	-0.68	-0.77	-0.62	-0.71	-0.77	-0.75	-0.66	-0.76	-0.60	-0.68	-0.76	-0.73	-0.65	-0.73	-0.71	-0.64	-0.74	-0.66	-0.66	-0.60	-0.65	-0.65	-0.54	-0.63
TG	4.1	STMN1	0.72	0.75	0.67	0.62	0.93	0.88	0.65	-0.66	-0.74	-0.65	-0.66	-0.71	-0.65	-0.62	-0.76	-0.70	-0.66	-0.56	-0.63	-0.64	-0.68	-0.65	-0.65	-0.70	-0.61	-0.50	-0.72	-0.57	-0.76	-0.59	-0.59	-0.68	-0.64	-0.69	-0.69	-0.64	-0.67	-0.74	-0.64	-0.68	-0.71	-0.70	-0.71	-0.63	-0.67	-0.65	-0.60	-0.56	-0.60	-0.50	-0.64
TG	3.9	MCM10	0.76	0.77	0.71	0.66	0.93	0.87	0.71	-0.67	-0.70	-0.63	-0.64	-0.68	-0.63	-0.60	-0.72	-0.67	-0.69	-0.54	-0.63	-0.62	-0.73	-0.70	-0.63	-0.74	-0.57	-0.51	-0.72	-0.62	-0.73	-0.56	-0.64	-0.72	-0.69	-0.70	-0.70	-0.60	-0.68	-0.73	-0.73	-0.71	-0.74	-0.69	-0.68	-0.64	-0.73	-0.70	-0.66	-0.60	-0.63	-0.55	-0.64
TG	3.8	EZH2	0.78	0.72	0.65	0.62	0.93	0.81	0.66	-0.64	-0.67	-0.62	-0.64	-0.65	-0.65	-0.64	-0.70	-0.67	-0.68	-0.56	-0.62	-0.64	-0.73	-0.70	-0.61	-0.73	-0.53	-0.51	-0.72	-0.63	-0.75	-0.59	-0.65	-0.73	-0.69	-0.69	-0.74	-0.57	-0.65	-0.74	-0.66	-0.67	-0.72	-0.70	-0.65	-0.70	-0.63	-0.68	-0.59	-0.62	-0.63	-0.52	-0.60
TG	3.7	RFC4	0.73	0.73	0.67	0.62	0.91	0.84	0.71	-0.65	-0.71	-0.63	-0.66	-0.68	-0.64	-0.61	-0.72	-0.69	-0.73	-0.59	-0.62	-0.63	-0.74	-0.73	-0.63	-0.70	-0.59	-0.52	-0.71	-0.64	-0.73	-0.56	-0.63	-0.74	-0.65	-0.70	-0.76	-0.65	-0.60	-0.73	-0.64	-0.65	-0.71	-0.72	-0.69	-0.72	-0.63	-0.68	-0.59	-0.62	-0.64	-0.57	-0.62
TG	3.6	GTSE1	0.79	0.74	0.65	0.61	0.92	0.80	0.67	-0.73	-0.69	-0.66	-0.67	-0.68	-0.68	-0.68	-0.69	-0.68	-0.71	-0.61	-0.67	-0.66	-0.76	-0.74	-0.63	-0.78	-0.53	-0.56	-0.73	-0.68	-0.79	-0.63	-0.70	-0.75	-0.73	-0.72	-0.74	-0.57	-0.69	-0.79	-0.71	-0.73	-0.75	-0.71	-0.67	-0.71	-0.76	-0.71	-0.68	-0.63	-0.66	-0.57	-0.68
TG	3.5	FANCD2	0.77	0.76	0.70	0.66	0.92	0.86	0.71	-0.67	-0.70	-0.62	-0.65	-0.68	-0.63	-0.62	-0.73	-0.67	-0.70	-0.57	-0.63	-0.62	-0.75	-0.73	-0.62	-0.77	-0.56	-0.50	-0.68	-0.65	-0.75	-0.56	-0.67	-0.74	-0.73	-0.69	-0.72	-0.60	-0.68	-0.75	-0.71	-0.65	-0.78	-0.72	-0.66	-0.70	-0.68	-0.68	-0.59	-0.63	-0.65	-0.56	-0.63
	3.3	SPDL1	0.63	0.80	0.63	0.59	0.91	0.82	0.60	-0.72	-0.77	-0.71	-0.68	-0.74	-0.70	-0.67	-0.76	-0.74	-0.61	-0.62	-0.68	-0.68	-0.65	-0.66	-0.69	-0.68	-0.64	-0.59	-0.64	-0.58	-0.62	-0.62	-0.57	-0.65	-0.66	-0.62	-0.64	-0.64	-0.63	-0.60	-0.63	-0.68	-0.61	-0.60	-0.76	-0.55	-0.63	-0.57	-0.50	-0.53	-0.63	-0.42	-0.70
TG	2.9	FAM72B	0.80	0.74	0.64	0.60	0.92	0.86	0.65	-0.68	-0.70	-0.62	-0.62	-0.67	-0.65	-0.63	-0.71	-0.67	-0.70	-0.55	-0.64	-0.63	-0.73	-0.72	-0.62	-0.78	-0.55	-0.51	-0.74	-0.62	-0.80	-0.58	-0.64	-0.73	-0.70	-0.73	-0.71	-0.58	-0.69	-0.80	-0.69	-0.81	-0.77	-0.72	-0.67	-0.69	-0.74	-0.69	-0.68	-0.57	-0.68	-0.61	-0.65
TG	2.5	BOLA2	0.73	0.97	0.69	0.69	0.83	0.71	0.67	-0.69	-0.72	-0.68	-0.69	-0.68	-0.65	-0.72	-0.73	-0.70	-0.60	-0.70	-0.69	-0.69	-0.67	-0.68	-0.75	-0.63	-0.61	-0.72	-0.62	-0.71	-0.64	-0.67	-0.67	-0.72	-0.70	-0.67	-0.62	-0.76	-0.68	-0.77	-0.70	-0.65	-0.62	-0.71	-0.64	-0.69	-0.67	-0.64	[illegible]	-0.64	-0.64	-0.45	-0.70
TG	2.3	HMGA1	0.91	0.64	0.59	0.58	0.66	0.64	0.65	-0.56	-0.45	-0.42	-0.39	-0.41	-0.45	-0.45	-0.48	-0.44	-0.57	-0.33	-0.45	-0.43	-0.63	-0.65	-0.40	-0.69	-0.32	-0.36	-0.64	-0.54	-0.70	-0.38	-0.61	-0.62	-0.62	-0.67	-0.61	-0.29	-0.71	-0.70	-0.66	-0.72	-0.69	-0.68	-0.46	-0.59	-0.71	-0.62	-0.67	-0.58	-0.54	-0.54	-0.52
TG	-2.0	AX747124	-0.66	-0.48	-0.50	-0.47	-0.60	-0.60	-0.60	0.55	0.43	0.36	0.41	0.37	0.44	0.45	0.36	0.37	0.52	0.41	0.48	0.44	0.54	0.49	0.39	0.55	0.33	0.40	0.69	0.40	0.74	0.40	0.45	0.54	0.46	0.75	0.55	0.35	0.47	0.75	0.53	0.70	0.71	0.55	0.42	0.67	0.62	0.78	0.64	0.52	0.74	0.93	0.48
TG OS	-2.1	ABCG1	-0.63	-0.47	-0.49	-0.46	-0.57	-0.61	-0.61	0.54	0.41	0.32	0.36	0.34	0.40	0.40	0.31	0.32	0.45	0.38	0.44	0.39	0.45	0.41	0.35	0.49	0.31	0.37	0.67	0.31	0.74	0.35	0.39	0.46	0.41	0.74	0.49	0.33	0.41	0.75	0.49	0.71	0.72	0.55	0.40	0.59	0.60	0.78	0.65	0.49	0.76	0.97	0.48
TG	-2.1	DNAJC25	-0.84	-0.75	-0.72	-0.71	-0.77	-0.68	-0.75	0.65	0.59	0.56	0.55	0.55	0.58	0.59	0.60	0.56	0.63	0.47	0.59	0.57	0.67	0.66	0.53	0.79	0.43	0.52	0.73	0.58	0.83	0.56	0.69	0.68	0.74	0.75	0.65	0.42	0.92	0.79	0.79	0.68	0.74	0.72	0.59	0.67	0.76	0.72	0.63	0.70	0.62	0.53	0.61
TP TG	-2.2	CYP4F2	-0.75	-0.59	-0.61	-0.59	-0.71	-0.68	-0.66	0.53	0.47	0.38	0.44	0.42	0.42	0.44	0.45	0.41	0.59	0.37	0.49	0.43	0.62	0.56	0.39	0.69	0.33	0.36	0.71	0.45	0.85	0.41	0.54	0.63	0.59	0.69	0.63	0.38	0.57	0.86	0.63	0.68	0.90	0.70	0.45	0.69	0.71	0.72	0.63	0.65	0.77	0.78	0.45
TG	-2.2	APCDD1L	-0.65	-0.44	-0.51	-0.47	-0.55	-0.57	-0.61	0.54	0.39	0.32	0.37	0.33	0.40	0.41	0.31	0.32	0.48	0.39	0.45	0.40	0.51	0.46	0.35	0.53	0.28	0.37	0.67	0.36	0.76	0.36	0.43	0.51	0.45	0.74	0.52	0.31	0.44	0.76	0.53	0.69	0.75	0.55	0.37	0.64	0.64	0.78	0.68	0.51	0.76	0.98	0.47
	-2.3	PRRT1	-0.39	-0.52	-0.53	-0.45	-0.62	-0.48	-0.49	0.85	0.78	0.85	0.81	0.80	0.88	0.85	0.66	0.81	0.50	0.91	0.82	0.88	0.46	0.49	0.87	0.47	0.78	0.84	0.49	0.57	0.46	0.79	0.43	0.44	0.47	0.53	0.44	0.71	0.46	0.45	0.42	0.55	0.37	0.31	0.79	0.45	0.55	0.47	0.50	0.28	0.46	0.38	0.85
TP TG	-2.3	CYP4F8	-0.73	-0.62	-0.56	-0.54	-0.71	-0.70	-0.64	0.60	0.52	0.43	0.47	0.47	0.48	0.49	0.49	0.45	0.62	0.42	0.53	0.46	0.63	0.60	0.42	0.69	0.36	0.42	0.67	0.49	0.85	0.45	0.58	0.62	0.60	0.66	0.63	0.39	0.59	0.87	0.69	0.73	0.91	0.71	0.49	0.65	0.73	0.68	0.68	0.66	0.76	0.75	0.54
TG	-2.5	ALDH6A1	-0.76	-0.74	-0.75	-0.73	-0.73	-0.68	-0.77	0.72	0.60	0.59	0.62	0.59	0.61	0.62	0.59	0.58	0.69	0.55	0.65	0.61	0.75	0.72	0.58	0.81	0.48	0.57	0.69	0.66	0.79	0.57	0.73	0.72	0.77	0.75	0.72	0.47	0.78	0.80	0.90	0.67	0.80	0.69	0.60	0.74	0.81	0.75	0.71	0.72	0.71	0.64	0.68
TP TG	-2.5	CYP4F3	-0.74	-0.60	-0.59	-0.58	-0.69	-0.69	-0.65	0.53	0.44	0.36	0.41	0.39	0.40	0.41	0.42	0.38	0.58	0.36	0.46	0.40	0.61	0.56	0.37	0.65	0.32	0.33	0.68	0.46	0.83	0.37	0.54	0.60	0.55	0.70	0.62	0.35	0.57	0.86	0.63	0.68	0.95	0.66	0.43	0.67	0.71	0.73	0.62	0.62	0.77	0.81	0.46
	-2.6	C16orf95	-0.32	-0.43	-0.43	-0.33	-0.56	-0.39	-0.42	0.78	0.72	0.77	0.78	0.76	0.82	0.81	0.62	0.75	0.56	0.91	0.76	0.82	0.48	0.49	0.79	0.44	0.68	0.77	0.46	0.57	0.47	0.78	0.45	0.46	0.45	0.45	0.47	0.72	0.35	0.46	0.40	0.47	0.36	0.35	0.69	0.52	0.46	0.43	0.46	0.34	0.43	0.39	0.75
	-2.8	RUFY4	-0.34	-0.57	-0.53	-0.48	-0.62	-0.58	-0.51	0.60	0.89	0.78	0.77	0.87	0.69	0.58	0.87	0.83	0.49	0.64	0.66	0.68	0.42	0.42	0.80	0.43	0.89	0.61	0.51	0.38	0.39	0.62	0.33	0.41	0.41	0.47	0.37	0.92	0.43	0.37	0.36	0.44	0.32	0.43	0.87	0.38	0.39	0.41	0.30	0.29	0.38	0.21	0.68
TI	-3.1	OR6Q1	-0.62	-0.58	-0.45	-0.38	-0.62	-0.50	-0.53	0.59	0.58	0.60	0.64	0.59	0.61	0.60	0.59	0.63	0.93	0.58	0.60	0.62	0.86	0.88	0.59	0.61	0.48	0.55	0.53	0.87	0.62	0.58	0.87	0.86	0.62	0.50	0.86	0.55	0.50	0.62	0.71	0.44	0.54	0.67	0.54	0.76	0.55	0.50	0.58	0.68	0.47	0.39	0.61
TG	-3.2	CTH	-0.71	-0.59	-0.63	-0.60	-0.64	-0.56	-0.61	0.74	0.57	0.63	0.62	0.58	0.68	0.70	0.54	0.59	0.60	0.59	0.71	0.68	0.66	0.66	0.61	0.72	0.42	0.64	0.74	0.61	0.73	0.66	0.66	0.68	0.76	0.63	0.41	0.69	0.74	0.75	0.67	0.68	0.63	0.58	0.67	[illegible]	0.94	0.75	0.76	0.60	0.61	0.58	0.72
TG	-3.5	FETUB	-0.69	-0.59	-0.59	-0.58	-0.66	-0.62	-0.66	0.60	0.45	0.40	0.43	0.42	0.46	0.46	0.37	0.41	0.51	0.41	0.49	0.45	0.55	0.51	0.42	0.60	0.36	0.44	0.64	0.46	0.76	0.39	0.51	0.54	0.53	0.71	0.55	0.32	0.59	0.79	0.61	0.70	0.80	0.57	0.46	0.60	0.69	0.73	0.63	0.56	0.92	0.81	0.53
	-4.0	HRG	-0.68	-0.52	-0.54	-0.50	-0.57	-0.59	-0.61	0.61	0.43	0.39	0.41	0.38	0.44	0.45	0.36	0.37	0.51	0.43	0.48	0.45	0.55	0.54	0.41	0.55	0.35	0.41	0.67	0.47	0.75	0.36	0.48	0.55	0.47	0.78	0.55	0.32	0.50	0.78	0.59	0.73	0.78	0.58	0.42	0.64	0.71	0.78	0.76	0.55	0.71	0.90	0.52
TG	-5.4	TAT	-0.72	-0.62	-0.54	-0.50	-0.66	-0.64	-0.61	0.69	0.57	0.54	0.55	0.54	0.60	0.61	0.52	0.55	0.62	0.57	0.63	0.60	0.60	0.61	0.55	0.61	0.45	0.57	0.73	0.53	0.78	0.54	0.55	0.60	0.53	0.79	0.59	0.44	0.56	0.82	0.66	0.77	0.73	0.67	0.55	0.69	0.81	0.80	0.90	0.62	0.69	0.78	0.65
TG	-6.0	CYP2B6	-0.77	-0.71	-0.61	-0.58	-0.77	-0.74	-0.63	0.74	0.65	0.59	0.57	0.59	0.64	0.63	0.58	0.59	0.61	0.56	0.67	0.61	0.65	0.63	0.58	0.75	0.50	0.58	0.79	0.53	0.80	0.60	0.58	0.63	0.67	0.78	0.62	0.47	0.72	0.79	0.71	0.91	0.76	0.65	0.64	0.64	0.78	0.76	0.75	0.58	0.72	0.71	0.70
	-14.1	AP000867.1	-0.55	-0.72	-0.63	-0.60	-0.73	-0.70	-0.62	0.60	0.87	0.76	0.72	0.84	0.68	0.56	0.91	0.83	0.59	0.55	0.62	0.67	0.53	0.55	0.78	0.55	0.85	0.53	0.59	0.46	0.56	0.57	0.43	0.54	0.48	0.59	0.49	0.84	0.58	0.55	0.48	0.53	0.44	0.60	0.85	0.49	0.52	0.51	0.43	0.44	0.45	0.29	0.65

LncRNAs with their clinical association are shown in light orange background. mRNAs with their clinical association are shown in light blue background. TG: Tumor Grade, TC: Tumor Capsule, TI: Tumor Invasion, TP: Tumor properties, OS: Overall Survival.

TableS1b: Correlation between tumor invasion specific lncRNAs and mRNAs

Tumor Invasion		Clinical association<br>FC	TI<br>-2.91	TI<br>-2.79	TI<br>-3.86	TI<br>-3.33	TI<br>-2.51	TI<br>-6.71	TI<br>-6.42	TI<br>-5.98	TI<br>-3.79	TI<br>-3.62
**Clinical association**	**FC**	**Gene Symbol**	**AC124861.1**	**G028162**	**uc.325**	**G047015**	**RP11-316P17.2**	**MT1DP_NR_027781**	**MT1IP_NR_003669**	**MT1DP_NR_003658**	**G078205**	**MT1IP_NR_104046**
TI	-5.8	**MT1M**	0.62	0.64	0.67	0.65	0.66	0.99	0.99	0.99	0.66	0.97
TI	-6.3	**MT2A**	0.62	0.64	0.68	0.64	0.63	1.00	1.00	0.99	0.66	0.95
TI	-6.6	**MT1IP**	0.59	0.63	0.67	0.59	0.60	0.99	0.99	0.97	0.64	0.93
TI	-6.7	**MT1H**	0.64	0.66	0.68	0.67	0.66	1.00	0.99	0.99	0.68	0.96
TI	-7.4	**MT3**	0.63	0.64	0.68	0.66	0.65	1.00	0.99	0.99	0.66	0.94
TI	-8.2	**MT1E**	0.56	0.61	0.61	0.57	0.59	0.96	0.95	0.94	0.61	0.94
TI	-9.3	**MT4**	0.61	0.64	0.67	0.62	0.62	0.99	0.99	0.98	0.65	0.95
	-5.0	**SERPINB12**	0.90	0.85	0.67	0.95	0.92	0.70	0.70	0.72	0.85	0.68
TG	-8.5	**MT1X**	0.58	0.60	0.67	0.60	0.57	0.93	0.94	0.93	0.62	0.86
TC TI	-17.2	**MT1F**	0.56	0.61	0.65	0.55	0.59	0.91	0.92	0.89	0.64	0.92
	-2.9	**AX747550**	0.90	0.92	0.60	0.87	0.82	0.63	0.64	0.65	0.81	0.60
	-3.5	**RP11-362K2.2**	0.89	0.88	0.64	0.90	0.85	0.66	0.66	0.66	0.87	0.66
	-3.9	**MYT1L**	0.87	0.87	0.65	0.83	0.76	0.65	0.66	0.65	0.92	0.64
TC TI	-4.0	**AK307150**	0.64	0.66	0.90	0.65	0.60	0.65	0.66	0.66	0.65	0.60
	-5.4	**GPR119**	0.86	0.80	0.62	0.94	0.89	0.63	0.62	0.66	0.76	0.60

LncRNAs with their clinical association are shown in light orange background. mRNAs with their clinical association are shown in light blue background. TG: Tumor Grade, TC: Tumor Capsule, TI: Tumor Invasion, TP: Tumor properties, OS: Overall Survival.

TableS1c: Correlation between tumor capsule specific lncRNAs and mRNAs

Tumor Capsule		Clinical association FC	TC 2.08	TC -5.53	TC -2.43	TC -3.31	TC -8.11	TC -6.42	TC -5.89	TC -3.15	TC -5.86
**Clinical association**	**FC**	**Gene Symbol**	**DCAF13P3**	**G064039**	**G080075**	**UBA6-AS1**	**RP11-119J18.1**	**RP11-167H9.5**	**GSE61474_XLOC_060526**	**G018382**	**XLOC_008995**
TG	**-4.6**	**KLK2**	-0.61	0.97	0.92	0.95	0.95	0.94	0.93	0.86	0.92
	**-2.8**	**RUFY4**	-0.57	0.92	0.91	0.91	0.89	0.90	0.90	0.93	0.87
	**-20.0**	**HIST1H1A**	-0.70	0.97	0.85	0.92	0.94	0.91	0.91	0.80	0.93
	**-3.0**	**KIRREL3**	-0.50	0.92	0.89	0.90	0.92	0.94	0.88	0.85	0.82
	**-3.1**	**KRTAP10-8**	-0.66	0.96	0.90	0.91	0.90	0.88	0.90	0.84	0.95
	**-4.2**	**NLRP9**	-0.65	0.96	0.87	0.90	0.92	0.94	0.89	0.82	0.87
	**-4.5**	**AK055785**	-0.64	0.95	0.90	0.91	0.90	0.89	0.92	0.84	0.94
	**-14.1**	**AP000867.1**	-0.68	0.96	0.86	0.92	0.92	0.86	0.91	0.79	0.97
	**-3.1**	**PRSS38**	-0.56	0.92	0.87	0.88	0.89	0.93	0.86	0.81	0.82
TG TC	**2.2**	**DCAF13**	0.91	-0.66	-0.55	-0.59	-0.59	-0.54	-0.54	-0.51	-0.64

LncRNAs with their clinical association are shown in light orange background. mRNAs with their clinical association are shown in light blue background. TG: Tumor Grade, TC: Tumor Capsule, TI: Tumor Invasion, TP: Tumor properties, OS: Overall Survival.

TableS1d: Correlation between overall survival specific lncRNAs and mRNAs

Overall survival		Clinical association FC	OS -2.29	OS -5.02	OS -2.34	OS -2.78
**Clinical association**	**FC**	**Gene Symbol**	**LINC01554**	**G019663**	**XLOC_006182**	**XLOC_007985**
	**-2.8**	**RUFY4**	0.18	0.93	0.41	0.70
OS	**-2.9**	**C5orf27**	0.95	0.21	0.28	0.46
OS	**-3.0**	**KRTAP4-1**	0.32	0.52	0.94	0.62
	**-3.1**	**KRTAP10-8**	0.20	0.91	0.39	0.70
	**-4.5**	**AK055785**	0.23	0.92	0.40	0.70
TG	**-4.6**	**KLK2**	0.21	0.94	0.47	0.73
	**-5.0**	**SERPINB12**	0.45	0.57	0.54	0.91

LncRNAs with their clinical association are shown in light orange background. mRNAs with their clinical association are shown in light blue background. TG: Tumor Grade, TC: Tumor Capsule, TI: Tumor Invasion, TP: Tumor properties, OS: Overall Survival.

TableS1e: Correlations of tumor properties specific lncRNAs and mRNAs

**Tumor properties**		**Clinical association**	TP	TP
		**FC**	**-2.09**	**-3.86**
**Clinical association**	**FC**	**Gene Symbol**	**XLOC_010739**	**G015949**
	**-2.2**	**PRRT3**	0.96	0.58
TP	**-2.2**	**AL590560.1**	0.95	0.60
TP	**-2.4**	**AC114783.1**	0.95	0.60
TP OS	**-2.6**	**AZGP1**	0.52	0.94

LncRNAs with their clinical association are shown in light orange background. mRNAs with their clinical association are shown in light blue background. TG: Tumor Grade, TC: Tumor Capsule, TI: Tumor Invasion, TP: Tumor properties, OS: Overall Survival.

Table S2: Pathways associated with differentially expressed lncRNAs, clinically relevant lncRNAs, potential master regulators and lncRNAs specific to clinical phenotypes.

Clinical Phenotypes	lncRNAs	Correlated genes	Number of correlated/ regulated genes
	28 upregulated lncRNAs, 311 downregulated lncRNAs	Correlated genes	108 upregulated mRNAs, 161 downregulated mRNAs
	17 upregulated lncRNAs, 87 downregulated lncRNAs	Clinically relevant lncRNAs Correlated genes	100 upregulated mRNAs, 72 downregulated mRNAs
TG TC	1) G073851#, 2) PTTG3P#, 3) RACGAP1P, 4) GSE61474_XLOC_040880##, 5) CTD-2267D19.6###	Potential master regulator (PMR) networks	Poor Prognosis (91 genes)
TI	1) MT1DP_NR_027781*, 2) MT1DP_NR_003658*, 3) MT1IP_NR_003669*, 4) MT1IP_NR_104046*		Good Prognosis (8 genes)
TG TC	GSE61474_XLOC_040880##	PMR GSE regulated genes	10 upregulated mRNAs, 47 downregulated mRNAs
TG TC	CTD-2267D19.6###	PMR CTD regulated genes	6 upregulated mRNAs, 45 downregulated mRNAs
TG	1) G073851# 2) PTTG3P# 3) GSE61474_XLOC_065503 4) RP11-708J19.2 5) ANXA2P1 6) ANXA2P2 7) ANXA2P3	Tumor grade specific lncRNAs correlated genes	Poor Prognosis (91 genes)
TG	44 lncRNAs		Good Prognosis (40 genes)
TI	1) MT1DP_NR_027781* 2) MT1DP_NR_003658* 3) MT1IP_NR_003669* 4) MT1IP_NR_104046* 5) AC124861.1 6) G028162 7) uc.325 8) G047015 9) RP11-316P17.2 10) G078205	Invasion specific lncRNAs correlated genes	Good Prognosis (15 genes)
TC	1) G064039 2) G080075 3) UBA6-AS1 4) RP11-119J18.1 5) RP11-167H9.5 6) GSE61474_XLOC_060526 7) G018382 8) XLOC_008995	Encapsulation-specific lncRNAs correlated genes	Good Prognosis (9 genes)

Pathways (column headings):

Pathways	Group	Pathway
Cell cycle		Cell cycle; Nonhomologous End joining; E3 ubiquitin ligases; Packaging of telemere ends; Mitosis; Cell cycle checkpoints; G2/M checkpoints; Mitotic spindle checkpoint; MAD2 inhibitory signal; Amplification of signal from the kinetochores; Resolution of sister chromatids; Separation of sister chromatids; M phase; Condensation of prophase chromosomes; Prometaphase; Metaphase and anaphase; Anaphase
Signaling pathway		Signal transduction; Activation of formins; Signaling by Rho GTPases; Rho GTPases effectors
Transcription Regulation	Epigenetic mechanism	DNA methylation; PRC2 methylates histones and DNA
	Transcription pathways	Generic transcription pathway; RNA polymerase I promoter opening; RNA polymerase I chain elongation; RNA polymerase II transcription
	Regulation of rRNA expression	rRNA regulation by SIRT1; rRNA regulation by ERCC6 and EHMT2
Metallothionein		Cellular responses to external stimuli; Metallothioneins bind metals; Response to metal ions
Metabolism	Metabolism	Biological oxidations; Metabolism; Acylglycerol biosynthetic process; Regulation of cellular carbohydrates
	Drug metabolism	Phase I - Functionalization of compounds; Cytochrome P450; Drug metabolism; Xenobiotic metabolism
	Carboxylic acid metabolism	Carboxylic acid metabolism; Carboxylic acid biosynthesis
	Amino acid metabolism	Metabolism of amino acids and derivatives; Amino acid catabolism; Tyrosine metabolism; Cysteine and methionine metabolism
	Fatty acid metabolism	Fatty acids; Fatty acids metabolism; Fatty acid degradation; Eicosanoids; Arachidonic acid metabolism; Synthesis of Leukotrienes (LT) and Eoxins (EX); Synthesis of (16-20)-HETE
	Glucose metabolism	Starch and sucrose metabolism; Fructose metabolism; Fructose and mannose metabolism; Glycolysis; Gluconeogenesis; Pentose phosphate pathway
	Proteoglycan metabolism	Glycosaminoglycan metabolism; Proteoglycan metabolism; Chondroitin sulfate catabolism; Dermatan sulfate biosynthesis; Peptide cross-linking
Others		Plasma lipoprotein remodeling; COPI-dependent Golgi-to-ER retrograde traffic; Endocytosis; Serine-type peptidase activity

Details of potential master regulators are in orange box. Experimentally demonstrated pathways are highlighted in blue box. Red box: Upregulated; Green box: Downregulated; TG: Tumor Grade; TC: Tumor Capsule; TI: Tumor Invasion. LncRNAs that are repeated in different rows are labeled with specific symbols next to the lncRNA names.

Table S3: Clinical characteristics of 49 HCC patients

Category	Clinical characteristics	Good characteristics	Poor characteristics	Data Not Available
Tumor properties	Tumor size	**< 5 cm**	**≥ 5 cm**	
		22	27	
	Vascular invasion	**Absent**	**Present**	
		27	22	
	Tumor stage	**Stage 1 or 2**	**Stage 3 or 4**	
		37	12	
Tumor grade	Tumor grade	**Grade 1 or 2**	**Grade 3 or 4**	
		20	29	
Tumor capsule	Encapsulation	**Present**	**Absent**	
		33	16	
	Degree of encapsulation	**Complete**	**Incomplete**	
		15	18	16
Tumor invasion	Tumor invasion	**Absent**	**Present**	
		18	26	5
Overall survival status	Overall survival status	**Alive**	**Deceased**	
		32	7	10

The 8 clinical characteristics are divided into 5 categories. Within each clinical characteristics, they are further classified into good characteristics and poor characteristics.

<u>Table S4: Primer sequences used for PCR/Real time RT-PCR reactions</u>

Gene name	Forward primer sequences (5' to 3')	Reverse primer sequences (5' to 3')	Purpose
Actin	ATGTTTGAGACCTTCACACC	AGGTAGTCAGTCAGGTCCCGGCC	PCR / Real time RT-PCR to detect expression in cell lines and patient samples
GSE61474_XLOC_040880 (lncRNA-GSE)	TTCCCCTCCCAACAGTCGAG	AGTGGACCGAAAGCTCAGGAA	
CTD-2267D16.3 (lncRNA-CTD)	GAAGGTGATGGAGTGTGTGTCT	TCTTTCCATGGCAACACCCAA	
CTD-2267D16.3 (lncRNA-CTD)	**GGTG**<u>GAATTC</u>CTACTTTGAAAGGGGATGCTTGAGG	**ATAAGAAT**<u>GCGGCCGC</u>TGGGGTTGAAACACACAATTTATTTTG	Cloning PCR

Underline sequences: Restriction enzyme (RE) sites. RE site at forward primer: *EcoRI* (5'-GAATTC-3'); RE site at reverse primer: *NotI* (5'-GCGGCCGC-3')

Bold sequences: Additional bases to facilitate efficient cleavage.

Table S5: cDNA sequences of lncRNA-GSE and lncRNA-CTD

LncRNAs	cDNA sequences
lncRNA-GSE (1,225bp)	>GSE61474_TCONS_00220963<br>CTCCACCCCAGCCGAACATCCCTGCAATGGCTTCATCCATCCTGACAAATAGTAACAAGCCACT<br>AGCACTTCTTGCCCCACACCTGTTTCCCCAGGAGACAGCATGGCTTCTCAGGAGCGTGTGCTCC<br>TTCAGAGCGGCTCTGTCTGCTCTCTCGCAGGGCGCTCCCAGCACCCAGAGCTGCGACCTGTCCT<br>TAACCAAGGGATCCGCTGAGCCCCCGGCTCCTCTTCTGTGTTTGCCTTGTCTGTGGGGCCTGTG<br>ATGCTCTCCGTCCGTGGCTTGACCCCCACCCATGAAAATGCTTCAGCACTTTCTCAGGAAAGAC<br>GCATGCCCTGTGTCCCCACTGGGTCCCATCCCCAGCCCCACCTTTAGCCTTGACTCAGATGCAC<br>AGAGATGCAGCTGTGGGTGGGCTCACCAATGTCCACACGCACCCCAGCCTGAAGCACGGTGCTA<br>CAGACCTAGTAGGGGCTGCAGAGATATTTTAAGGGAAAAATATAATTTACAGCTTCTTACAATA<br>GTTTGAAGTTCGGAAAGAGTGAGATTGGGGAAAAAATAGAAGCAGTAGCGGCGGCACTGATTTT<br>ACCAGGCTCTCTATGGGCTGAATTTTAAAAACTTGCCTTTCAAAGGGACTTGGAACTTCTTCCC<br>AATTCATTGTGTTGCTACTTTGCAAACTCCACTTGATTCCTGAGCTCATCATGAATTCTATAAA<br>TTTTGTATTTTTACACCAGGGCCCTTTGAAGTTGTACCTTTAAAGAGCAAAAACAAAACCAAAG<br>TGACACTTTCTTTTTTTTTTTTTAATAGAAAAGCGTGTGTAGCTGTGACATAGTTCACATTCCAC<br>AACCACTGAAGGGTTGAAACATGAACTTGAAAGTAAATAGCTTTGTGGAGATTCACATCGAGGA<br>GCACTGTGCAGTCATTCAGAAGAATAAACTCCAGCTCTGCCCGGTGATGGCAGGGGTGTTAACA<br>ATGCAGTATTGAGCTCAAAAAGTAACTTGCTAAAAGAACACATGCCCCCACATGAAAACGCGAA<br>AGGTGTCAGAGGTAGCGTTTCTACCTGTGGGCCTGCGGAGAGCTACGTGGATCTTCCCCTCCCA<br>ACAGTCGAGAAGACTCGAGGCCGCCTCTGAGCACCTCCCCTGGCTCAGCGGAGTACAGTGCACC<br>GTCCCCACAGCTCCTTCCTGAGCTTTCGGTCCACTTTAAATCAATAGCAGAAGAATGTATTTGA<br>TTATCAGGC
lncRNA-CTD (1,453bp)	>ENST00000602403<br>ctactttgaaaggggatgcttgaggctgaggtagttttgagggaaagggggaagttctagtttg<br>atttgtttgggagattgctgtcctttcataactgaagcaaatgcttgaaatgtaaaccatgtgt<br>gatacgaatgatctgagagccagtgaccaagtctgcttttccctgagctctcctgctgccgcct<br>ctacctttctgacatggccttccatttggaacttgatatgtgtacaagtgatctgttggccact<br>gtttcattcatcctgaccactgtatccgtgtaatgtgatctgagctacaacaggtggtatggga<br>tagaggcaaggaaagcagagagagccctctgaaatgaacactacccactggtgtgtgtagcaca<br>tgatagtctgtaggcatgagtctgaatgttgttctcacactcctttactagagtattcgttccc<br>tctgaaggtagtttgaacatggaaacacagttctatacctagaaggttggaaagtgtctgggac<br>tataagggttaacatgcataattgcaaagattgtccgaaggctgatggaaagcagagactgaat<br>gggattgagaacagatgcgaagcttgatttaaattacattttattggatgctgcagccttaaga<br>gacgtgactgctttacagtttgtttccacactgtgggcagctgctgtctgttctgtgtccacag<br>taggatcctgcccaataaggagcagcctccccacctcattgtgtttgaggcttggcgccttcct<br>cttaactgtagggcttgagtcaggaacatggcttgactcgcagtggggctgctatgtatcctcc<br>ctggcttccagccaaaatcacattggtagattcaaaggggccaaatttctttcccctctatctt<br>tccctttcccctggttttggaaatagagttttctgtctactgatttgttagtttccttttcttc<br>tcccctcactgtcaatttctaggtcattgctgctcttaagactttagcagttggaacagggttg<br>gttctgtcaatgatgcatgaagcagacttagtgtccctgcttggcttctgctgcccttgtggga<br>gcaaaagctgatatatgtttgtcagtaaagtcttaagtgtaattcagactgctgaggaagaaag<br>ccctttccttgctggcttttctccctgaagctgagagcttcaggaaggtggtaggagttttagt<br>gggatgggatgggatgggcttgtggttagcagtttttgccaggatcataggctgcttcacttag<br>agtagtggcaaagatgctgacctacattcttccttttgaaggtgatggagtgtgtgtctatatc<br>ctggtttatctctctcccccaccccaaggaagctgattaagcttccatagagtgttgggtgttg<br>ccatggaaagagcatagacaaaataaattgtgtgtttcaacccca
